# Uncovering Human Tooth Marks in the Search for Dog Domestication: The Case of Coímbre Cave

**DOI:** 10.3390/ani15091319

**Published:** 2025-05-02

**Authors:** Idoia Claver, Verónica Estaca, María de Andrés-Herrero, Darío Herranz-Rodrigo, David Álvarez-Alonso, José Yravedra

**Affiliations:** 1 Department of Geodynamics, Stratigraphy and Palaeontology, Faculty of Geological Sciences, Complutense University of Madrid, Ciudad Universitaria, Edificio B, Calle Profesor Aranguren, 28040 Madrid, Spain; idclaver@ucm.es; 2 Department of Prehistory, Ancient History and Archaeology, Faculty of Geography and History, Complutense University of Madrid, Ciudad Universitaria, Edificio B, Calle Profesor Aranguren, 28040 Madrid, Spain; 3 Research Group “Quaternary Ecosystems”, Faculty of Geological Sciences, Complutense University of Madrid, Ciudad Universitaria, Edificio B, Calle Profesor Aranguren, 28040 Madrid, Spain; 4 Research Group “Arqueología Prehistórica”, Faculty of Geography and History, Complutense University of Madrid, Ciudad Universitaria, Edificio B, Calle Profesor Aranguren, 28040 Madrid, Spain; 5 Department of Prehistory and Archaeology, Faculty of Philosophy and Letters, University of Granada, 18071 Granada, Spain; 6 HUM1103—Grupo de Investigación Cuaternario y Evolución Humana en África y el Sur de Iberia, (CUATE), Universidad de Granada, 18071 Granada, Spain; 7 C.A.I. Archaeometry and Archaeological Analysis, Complutense University of Madrid, Ciudad Universitaria, Edificio B, Calle Profesor Aranguren, 28040 Madrid, Spain

**Keywords:** taphonomy, human tooth marks, Magdalenian, geometric morphometrics

## Abstract

The domestication of the dog (*Canis lupus familiaris*) is one of the oldest and most complex processes of interaction between humans and animals. This phenomenon may have begun sometime between 30 and 15 ky calBP. Archaeological and genetic studies have provided valuable insights into dog domestication, although the precise geographic location and origin of this process remain controversial and under debate. New methodologies, such as taphonomic analyses, offer opportunities to deepen our understanding of past human–dog interactions. In this context, the present study examines tooth marks found on some bone remains from the Upper Magdalenian site (15,500–13,200 cal BP) of Coímbre Cave (Peñamellera Alta, Asturias, Spain). The low incidence of carnivores at the site has raised the possibility that the tooth marks may have been produced by canids. However, a detailed taphonomic analysis combining geometric morphometrics with robust statistical methods—including MANOVA with post-hoc permutation tests—revealed that the marks identified at the site do not significantly differ from tooth marks produced by humans (*p* = 0.086). In contrast, tooth marks produced by other carnivores, such as *Canis lupus signatus* and *Canis lupus familiaris*, showed significant differences (*p* < 0.003). Although our study could not confirm the presence of domesticated dogs at the Magdalenian levels of Coímbre Cave, it has documented taphonomic processes that are rarely identified in the archaeological record. Furthermore, this study highlights the potential of tooth mark analysis as a key tool for future research on human–animal interactions in archaeological contexts.

## Simple Summary

This work delves into the analysis of tooth marks supposedly produced by carnivores at the Upper Paleolithic site of Coímbre Cave. This is a Magdalenian site that shows significant human activity, yet tooth marks are also present on the bones. The objective of this work is to identify which carnivore produced these marks. However, our results have been surprising because they have shown that these tooth marks were not made by carnivores but by humans. This is important because it shows that humans could sometimes also produce tooth marks on bones.

## 1. Introduction

The domestication of the dog (*Canis lupus familiaris*) represents one of the most significant biocultural processes in human history. This process has been the focus of numerous studies over recent decades [1,2,3,4,5,6], spanning various scientific fields such as evolutionary biology, ecology, genetics, ethology [4,6,7,8,9,10,11,12,13,14,15,16,17], stable isotope analysis [18], archaeology, and geometric morphometrics [19]. The vast number of studies dedicated to this subject reflects the importance of dog domestication as a key milestone in the coevolution between humans and canids, as has occurred with other animal species. What began as a speculative field has evolved into an interdisciplinary area of research, thanks to advances in scientific techniques that have considerably expanded our understanding of these human-driven processes.

It is estimated that the relationship between humans and dogs began over 15,000 years ago, based on genetic and archaeological evidence suggesting a complex and possibly multiple-origin domestication process [20,21]. Researchers such as Raymond and Lorna Coppinger, Pat Shipman, Greg Larson, and Ádám Miklósi have explored the origins of dog domestication, proposing that it was gradual and supported by natural selection. Genetic studies have shown that modern gray wolves (*Canis lupus*) and dogs (*Canis lupus familiaris*) originated from an extinct lineage of archaic wolves that lived between 40 and 20 ka cal BP. Therefore, both species share a now-extinct common ancestor.

Wolves predating the gray wolf displayed less aggressive behavior, which facilitated their proximity to humans. Attracted by human settlements and food waste, these more docile wolves made initial contact with humans—similar to the behavior of current species such as hyenas or lions in certain environments. This first contact led to a symbiotic relationship, in which the presence of wolves around human settlements provided mutual benefits. Over time, this association aided hunting, protection, and other aspects of human life, promoting a domestication process that was not necessarily intentional. This symbiosis strengthened the adaptive capabilities of *Homo sapiens*, facilitating their expansion and dominance. Furthermore, it has been demonstrated that dogs developed remarkable abilities for teamwork and understanding human cues—skills acquired through the domestication process [22,23,24].

Although genetic and archaeological research has made great strides, the exact geographic origin of dog domestication remains uncertain. Several regions have been proposed, including Europe [14], Asia [11], and the Middle East [25], but current evidence suggests a complex process that may have occurred in multiple locations simultaneously or in different periods [10]. Given this uncertainty, taphonomic studies have emerged as a potentially key tool for deepening our understanding of dog–human interactions in archaeological contexts. Taphonomy has become fundamental in reconstructing paleoecological and zooarchaeological processes, providing insight into how interactions between humans and animals developed over time [26,27].

Within this field, the analysis of tooth marks on bone remains has been widely used to identify consumption patterns, biological modifications, and prey processing [28,29]. Taphonomic analysis of tooth marks applied to the study of dog domestication allows researchers to infer the trophic and ecological impact of this process on the behavior of early domesticated dogs. Domestication has involved morphological, behavioral, and ecological changes in modern dogs, which may be reflected in how canids interacted with bone remains in different cultural contexts [20,21] or may have even influenced the way they bit bones, leaving different marks than those of their wild ancestors [30,31].

For this reason, the present study aims to analyze the origin of dog domestication through the taphonomic study of tooth marks, comparing those made by modern dogs and other animals with marks found in archaeological sites. This approach will help determine whether certain marks on bone remains can be attributed to domesticated dogs or other carnivores. Additionally, this analysis will enable a quantitative characterization of the variability in tooth marks and the comparison between modern animals and archaeological remains, providing key insights into the early stages of domestication.

In recent years, some studies have successfully differentiated tooth marks left by different carnivores with a high degree of accuracy [19,30,31,32,33,34]. Research using modern samples produced by dogs and wolves has been able to successfully distinguish between marks made by each species [30,31,34]. It is also important to consider the possibility of intermediate dog-wolf cases, which may exhibit dental characteristics of both species and produce tooth marks distinct from those typically attributed to either dogs or wolves [35]. Nevertheless, previous studies on canid tooth marks have shown sufficient sensitivity to detect differences even among various dog breeds [30,31,32]. Consequently, tooth marks resulting from intermediate dog-wolf stages could be classified as those produced by a carnivore other than a domestic dog or a wolf.

Following this approach, a previous study demonstrated that the activity of *Canis lupus familiaris* can be identified through tooth mark analysis in prehistoric archaeological contexts [36]. Building on that work, the present study applies our methodology to an archaeological sample from a Palaeolithic site—Coímbre Cave (Asturias, Spain). The objective of this research is to document the potential presence of dogs in the Iberian Magdalenian, as previously proposed by J. Altuna at the sites of Erralla and Urtiaga [37,38,39], and to evaluate the effectiveness of our methods in identifying processes associated with dog domestication.

The decision to employ this type of analysis stems from the fact that many prehistoric sites with well-preserved faunal remains show numerous bones bearing tooth marks. If domesticated canids were present at these sites, it is plausible that they were responsible for some of these marks. Identifying their origin could provide indirect evidence of dog activity in these Magdalenian contexts and thus contribute to documenting the domestication process.

## 2. Archaeological and Geological Context

**Figure 1:**
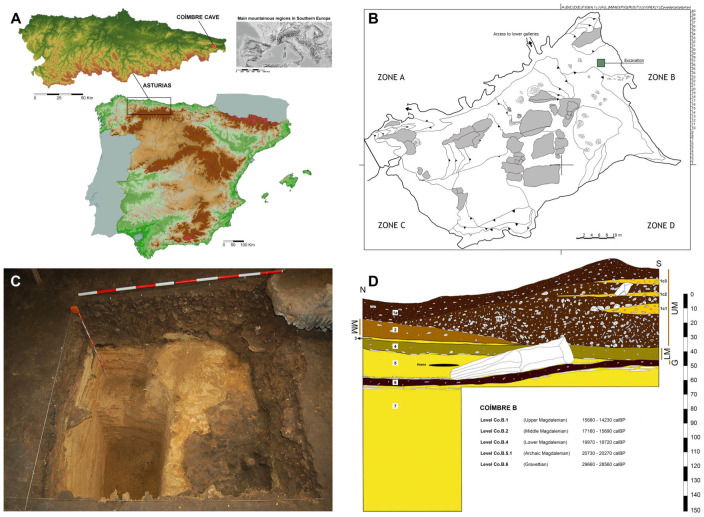
(**A**) Location of Coímbre Cave [42]; (**B**) Topographic plan of the main chamber of the cave with the excavation [41]; (**C**) Zone B of Coímbre Cave at the end of the 2012 excavation; (**D**) Stratigraphy of Zone B of Coímbre cave [41].

Coímbre Cave (Peñamellera Alta, Asturias) is located in the central-western area of the Cantabrian region, in the north of the Iberian Peninsula (Figure 1A) [40]. It is one of the most important Magdalenian sites in northern Iberia, excavated in recent years using modern archaeological methodologies [41].

From a geological point of view, the cave lies in the Besnes River valley (Figure 1B), within a context of Namurian and Westphalian deposits (Upper Carboniferous), belonging to the Barcaliente Formation [42,43,44].

Between 2009 and 2013, five excavation campaigns were carried out, focusing on the first level of the cave, known as the entrance hall. These included a 2 × 2 m excavation area (Figure 1C) in squares J-26, J-27, K-26, and K-27 (Figure 1D) [42].

The stratigraphy documented during the excavations allowed the identification of a sequence of sedimentary and occupational layers that reconstruct the natural, geomorphological, and human events that shaped the cave. Of particular interest is the level corresponding to the last phase of human activity in the cave, designated as Co.B.1 (Figure 1B) [42,45]:

Level Co.B.1 (Upper Magdalenian, 15,680–14,230 BP): This is the most complex level, subdivided into several layers. The uppermost layer, Co.B.1a, represents the final phase of human occupation. Below it, Co.B.1b corresponds to the initial phase of occupation, characterized by repeated cleaning of the cave floor to maintain a habitable space (sublevels Co.B.1c1, Co.B.1c2, and Co.B.1c3).

Research conducted at Coímbre Cave has provided valuable insights into human occupation in the region during the end of the Upper Paleolithic, as well as into the geological processes that have shaped the cave’s structure over time.

Taphonomic studies at the site revealed that Coímbre was a human habitat where various animals—especially goats, deer, and chamois—were exploited by humans [46,47]. These analyses also indicated the presence of systematic butchery processes, reflecting the development of different techniques such as skinning, defleshing, disarticulation, evisceration, and even tendon extraction [48].

## 3. Materials and Methods

Based on previous studies conducted at Coímbre Cave [46,47], which revealed a highly anthropized bone assemblage with numerous cut marks related to various butchering activities, as well as tooth marks initially interpreted as being produced by carnivores [47], the aim of this study is to analyze those tooth marks in order to identify the carnivore species responsible. Given the predominance of human activity in the fossil accumulation at Coímbre Cave, it is plausible that some of these marks may be attributed to dogs—especially considering that their presence has been documented at other Magdalenian sites in northern Iberia [37,38]. If confirmed, this would contribute valuable data to the current understanding of dog domestication during the Magdalenian in this region.

For this analysis, various high-resolution taphonomic techniques based on comparative statistical methods were used. These methods allow for the analysis of the marks found at Coímbre and their comparison with those produced by different extant animal species used as reference. Tooth marks left by carnivores can take several forms, notably tooth pit marks, which are circular depressions caused by the pressure of teeth on the cortical bone surface without perforating it; scores, which are scratches caused by dragging of teeth along the bone; punctures, which perforate the cortical surface; and holes, generated by digestive processes and characterized by the presence of cavities [28,49,50,51,52,53,54,55,56,57,58,59,60,61]. This study focuses specifically on tooth pit marks, as these marks exhibit low intraspecific variability. That is, within the same species, regardless of captivity status, sex, or the size of the bitten bone, these marks maintain a consistent morphology [30,32,33]. This contrasts with other types of tooth marks, such as tooth score marks, which may display higher variability [33]. These differences in tooth score marks between captive and free-ranging wolves may be related to variations in the number of marks and the bite intensity observed in captive individuals compared to their free-ranging counterparts [62,63,64]. However, these differences do not appear to affect tooth pits, which, according to previous studies, show no significant variability between the two populations [30,32,33].

For the study, 50 tooth pit marks located on the diaphyses of long bones from goat and chamois from Coímbre level Co.B.1 were analyzed and compared to a reference sample of tooth marks made by nine species from the following carnivore subfamilies, with 50 marks from each: Caninae (*Canis lupus signatus*, *Canis lupus familiaris*, *Lycaon pictus*, and *Vulpes vulpes*), Ursinae (*Ursus arctos*), Hyaeninae (*Crocuta crocuta*), and Pantherinae (*Panthera leo*, *P. onca*, and *P. pardus*) (as detailed in [19,32,33,65,66]). In the case of *Canis lupus familiaris*, 50 marks were obtained for each dog breed analyzed, including specimens of labrador retriever, mastiff, and rottweiler, and also 30 from an Irish setter and 28 from a boxer, as well as 50 from a mixed-breed dog previously described in [31]. Medium- and large-sized dogs were selected for the study due to their comparable body size to wolves, which typically weigh between 30 and 50 kg. This selection ensured accurate comparison between fossil marks and those left by similarly sized canids. In addition to carnivores, a new dataset of human tooth marks was also included. All marks used for the comparison between Co.B.1 and experimental samples were taken from diaphyses of long bones.

The experimental protocols for collecting tooth marks followed a detailed and systematic procedure. The samples were gathered in collaboration with various natural parks (Supplementary File S1). Diaphyses of long bones—mainly horse radii and tibiae—were offered to the animals, with some additional bones from smaller species such as roe deer, wild boar, and red deer (only in the wolf experiments). Previous research has shown that prey size does not significantly affect tooth mark morphology, so a wide range of bones could be used for comparison. The bones, previously partially skinned, were offered to the animals after feeding and left in the enclosures for several days, allowing interaction and the creation of tooth marks [31,33,61,66,67,68]. Afterward, the bones were retrieved and boiled in water, preserving the marks without using chemicals that might damage them. Once dried, the bones were prepared for further analysis [30,33,61,65,66].

**Figure 2:**
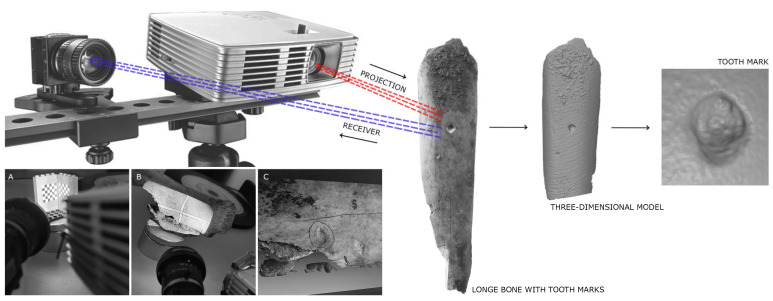
Modelling process using the DAVID SLS-2 3D scanner, showing how a three-dimensional model is obtained from an original specimen, followed by the extraction of the tooth mark. (**A**) 15 mm calibration marker; (**B**) Projected light from the projector; (**C**) Final virtual model of the cut mark, including texture.

After collecting the tooth marks, they were digitized in 3D to create a digital database and enable detailed sample analysis. Established methodologies (see [19,30,31,32,33,61,66]) were followed, starting with scanning the pits using a DAVID SLS-2 structured light 3D scanner (DAVID-Vision-Systems GmbH, 56070 Koblenz, Germany) (Figure 2) at the Archaeometry and Archaeological Analysis Unit of the Research Support Center for Earth Sciences and Archaeometry, Complutense University of Madrid. Macro lenses ranging from 2× to 10× magnification were used on both the camera and the projector. Before scanning, the equipment was calibrated using a reference template with markers at 15 mm intervals. Results were saved in .obj format and converted to .ply for landmark point processing.

**Figure 3:**
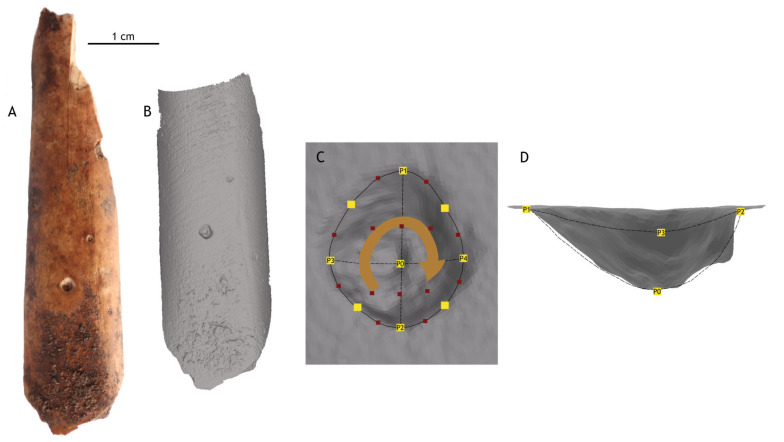
(**A**) Diaphysis fragment from Coímbre with a pit tooth mark. (**B**) Three-dimensional scan of the bone. (**C**) Occlusal view of the tooth mark showing the main anatomical landmarks (indicated with numbers) and the clockwise distribution of semi-landmarks. The distribution of semi-landmarks was generated using the Landmark software, which employs a patching function to automatically place equidistant semi-landmarks between the main reference points. (**D**) Cross-sectional view of the tooth mark.

Twenty-five landmarks were defined for each pit according to [32] (Figure 3). These included four perimeter-defining points, one indicating the deepest point, and twenty more distributed evenly across the morphology. Landmarks were recorded using Landmark software (version 3.0.0.6) and exported as “Raw Landmark Points”. 

For the digital database used during the statistical applications, 50 pits were randomly selected for each species, except for *Canis lupus familiaris*, for which 258 pits were selected. Specifically, the following number of pits were chosen for each breed: labrador retriever (50 pits), mastiff (50 pits), rottweiler (50 pits), mixed-breed dog (50 pits), Irish setter (30 pits), and boxer (28 pits).

This resulted in a database containing 658 pits. The Raw Landmark Points were stored in a text file in Morphologika format, which preserves landmark coordinates and three-dimensional matrices. This format is widely used in geometric morphometric studies [69,70].

Data analysis was carried out using the R programming language by loading the data with the function read.morphologika(file.choose( )), following these steps:

1. Generalized Procrustes Analysis (GPA): The shapes package was used to align the landmark points through translation, rotation, and scaling [71,72,73,74,75]. This analysis allowed for the extraction of morphological information from the samples, producing configurations based on shape (with scaling) and form (without scaling). As sample size does not influence comparative results, the decision was made to work with data in its original scale using the function procGPA(x$coords, scale = FALSE). Additionally, scaled analyses were conducted with procGPA(x$coords, scale = TRUE) to ensure that the results did not show significant variation.
2. Principal Component Analysis (PCA): Based on the GPA results, principal component (PCs) values were calculated and graphically represented using ggplot2. Confidence ellipses at 95% were included for each group (species or subfamily).
3. Ellipse Overlap Percentage Calculation: The sf and dplyr packages were used to assess the intersection between the confidence regions of the ellipses. For this purpose, the ellipse coordinates were extracted and converted into spatial geometry objects. The individual and intersection areas were then calculated using the functions st_area() and st_intersection(). Finally, the percentage of overlap was obtained using the following formulas:
  (1) $$\mathrm{Percentage}\;\mathrm{of}\;\mathrm{overlap}\;\mathrm{in}\;\mathrm{ellipse}\;1\;\mathrm{or}\;2=\frac{\mathrm{Area}\;\mathrm{of}\;\mathrm{intersection}}{\mathrm{Area}\;\mathrm{of}\;\mathrm{ellipse}\;1\;\mathrm{or}\;2}\times 100$$
4. Multivariate Analysis of Variance (MANOVA): Based on the PCs values obtained from the PCA, differences between groups across multiple dependent variables were evaluated using the function manova(). This analysis made it possible to determine whether there were significant morphological differences between the groups studied.
5. Post-hoc Pairwise Comparisons: In cases where the MANOVA indicated significant differences, pairwise comparisons were carried out using permutations via the function pairwise.perm.manova(), from the RVAideMemoire package. This non-parametric approach is robust to non-normal distributions and allows for the identification of which groups differ significantly. In this study, a more stringent threshold of *p* < 0.003 (equivalent to 3σ) was adopted instead of the traditional *p* < 0.05, to obtain more robust and reliable results indicating the absence of significant differences between the samples compared. The traditional *p*-value of 0.05 carries a high risk of type I errors (false positives), with up to a 28.9% probability of incorrectly accepting the alternative hypothesis when the null hypothesis is true. Conversely, using the more conservative threshold of *p* < 0.003 significantly reduces the risk of type I error, with only a 4.5% probability of making this type of error. Moreover, this stricter value increases the reliability of the results, reflecting stronger evidence in favor of the alternative hypothesis. In this way, using *p* < 0.003 ensures more robust conclusions and minimizes the likelihood of false positives, which is essential for ensuring the validity of findings in contexts with high variability or uncertainty (according to [76]). Before choosing which test to apply (Wilks’ lambda/ “Wilks” or Hotelling–Lawley Trace/ “Hotelling”), the Shapiro–Wilk test was performed using the function shapiro.test().
6. Procrustes ANOVA: To assess the relationship between shape and size (allometric analysis), the geomorph package [69] was used. A data frame was created from the GPA data using the function geomorph.data.frame, and then the function procD.lm(coords~ log(Csize) − sample) was applied, taking into account centroid size and the groups analyzed. Results were visualised graphically with ggplot2, using the function plotAllometry() with the methods “PredLine”, “RegScore”, “size.shape”, and “CAC”.

## 4. Results

### 4.1. Taphonomic Study of Tooth Marks

**Figure 4:**
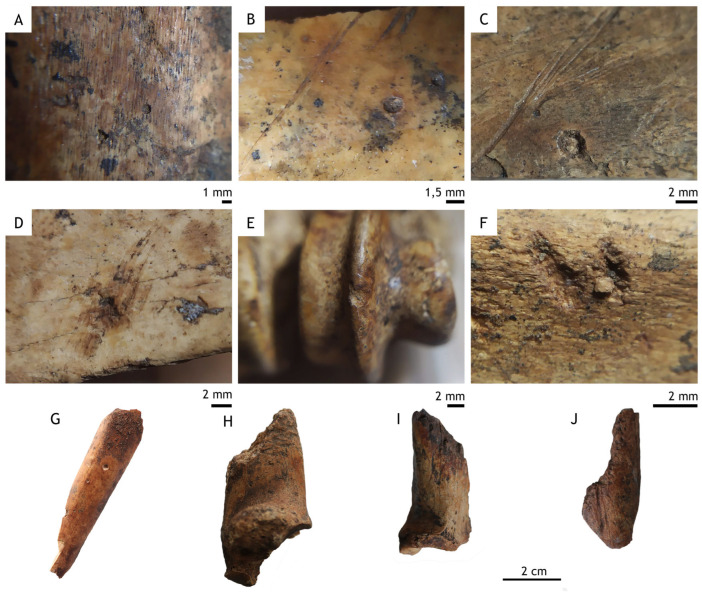
Example of bones with different types of tooth marks from the Coímbre Site, Zone B. (**A**): Small pit measuring 1 mm. (**B**,**C**): Pits no larger than 0.2 mm on long bones, adjacent to cut marks. (**D**): 2 mm pit overlying a cut mark. (**E**): 2 mm pit on the trochlea of a *Capra pyrenaica* metapodial. (**F**): Presence of scores ranging from 2 to 3 mm. (**G**): Fragment of a long bone with a pit. (**H**–**J**): Fragments of long bones with furrowing resulting in the loss of a large portion of the bone structure.

**Figure 5:**
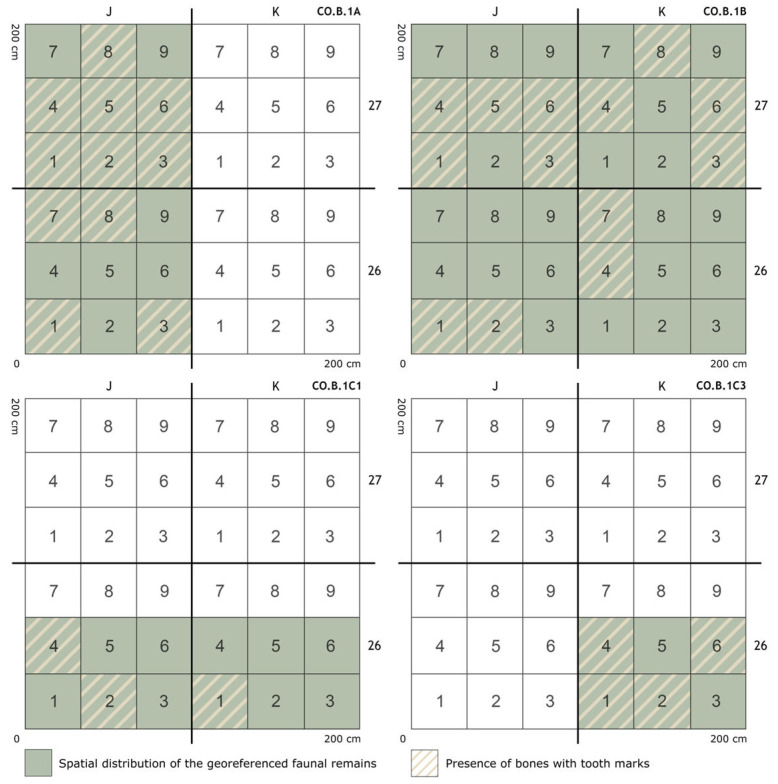
Spatial distribution for levels Co.B.1a, Co.B.1b, Co.B.1c1, and Co.B.1c3, in which tooth marks have been identified. The figure also shows the areas of the excavation grid where macrofaunal bone remains were recorded during the field seasons [40].

In relation to the stratigraphic levels previously described in the introduction, a statistical analysis was carried out on 50 tooth pit marks (Supplementary File S2) distributed across 36 diaphyses of long bone specimens belonging to goats, chamois, roe deer, red deer, and horses from various sub-levels of level 1 at Coímbre (Co.B.1a, Co.B.1b, Co.B.1c1, and Co.B.1c3), attributed to the Upper Magdalenian (Figure 4). The majority of the marks—specifically 27—were found on bones bearing a single pit, while six bones displayed two pits, two bones had three pits, and a single bone notably contained five pits. In all cases, the pits were located on the diaphyses of long bones, distributed across most of the excavation area (Figure 5).

In the statistical analyses, the tooth marks identified at the Coímbre Cave site were compared with those produced by canids, ursids, hyaenids, and felids—a total of 658 pits (408 from various types of canids, 50 from ursids, 50 from hyaenids, and 150 from felids). This comparison made it possible to establish a reference framework for identifying the taphonomic agent responsible for the marks observed at Coímbre.

However, since the main objective of the project is to investigate possible evidence of dog domestication, it was essential to rule out the possibility that the marks at Coímbre were the result of canid activity. To this end, comparisons were made with recent marks from *Vulpes vulpes*, *Canis lupus signatus*, and *Canis lupus familiaris* (a total of 408: 50 from Coímbre, 50 from foxes, 50 from wolves, and 258 from various dog breeds).

The results obtained through MANOVA revealed significant differences between the groups compared. Moreover, the Shapiro–Wilk test indicated that the values did not present homogeneity, and thus a post-hoc analysis using Wilks’ lambda was applied. In the statistical comparisons, all three species analysed yielded *p*-values of 0.001 (σ), suggesting that the tooth pit marks from the Coímbre Cave were not produced by canids.

**Figure 6:**
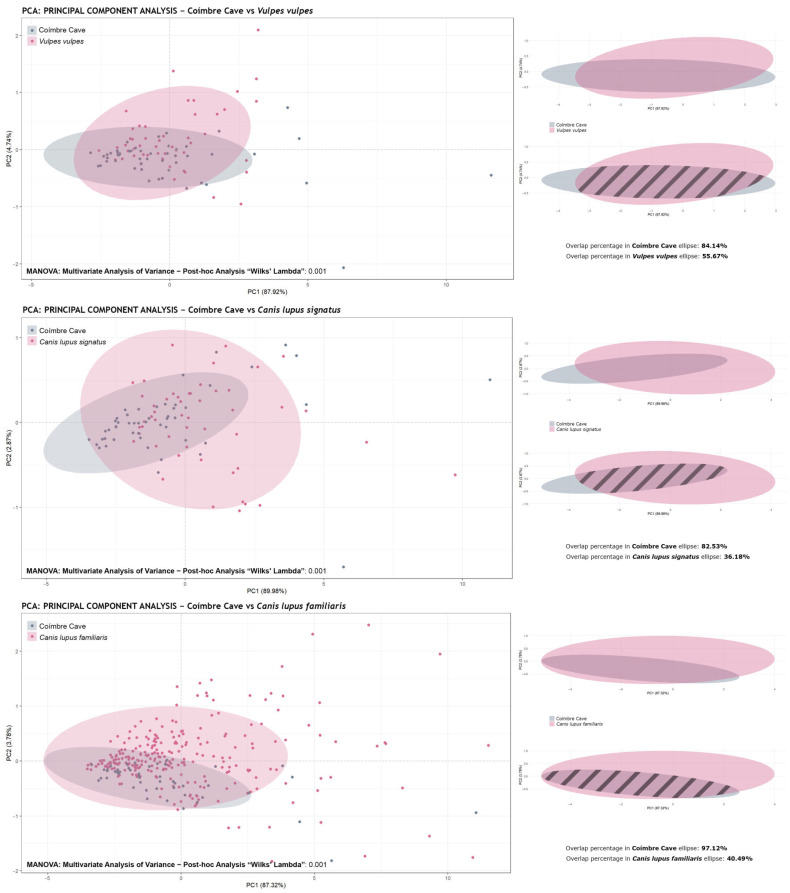
Principal component analysis (PCA) showing the PC1 and PC2 values for the tooth marks from the Coímbre Cave and the most representative canid species of the Iberian Peninsula (*Vulpes vulpes, Canis lupus signatus*, and *Canis lupus familiaris*). The data represented in the plot were not scaled (scale = FALSE), thus both the size and shape of the marks were taken into account. Additionally, post-hoc MANOVA results using permutations through Wilks’ lambda method are shown.

Consequently, it is not possible to statistically confirm the presence of domesticated canids at this site. Figure 6 shows the graphical representations of the principal component analysis (PCA) for each species compared with the Coímbre assemblage, as well as the results of the confidence ellipse overlap calculation. It was found that the Coímbre marks overlapped with 84.14% of the *Vulpes vulpes* ellipse, 82.53% of the *Canis lupus signatus* ellipse, and 97.12% of the *Canis familiaris* ellipse. The high percentages of overlap in the ellipses indicate that the species display similar morphological profiles in the multivariate space analyzed. This is due to the PCA being performed with the original sample size preserved (scale = FALSE), which highlights their general morphological proximity. However, the post-hoc MANOVA analyses reveal statistically significant differences between the samples, indicating that, despite the visual overlap, there are relevant variations in certain combinations of variables.

The significant differences found among the canid group prompted a broader comparison with all other species (a total of 708 pits, 50 from Coímbre and 658 from the database), in order to determine whether the taphonomic agent is represented within the database. The first analysis performed was a MANOVA, which revealed statistically significant differences between the groups. Furthermore, the Shapiro–Wilk test indicated that the values were not normally distributed.

As a result, a post-hoc analysis was conducted using the Wilks’ lambda method, and the *p*-values obtained are shown in Table 1. The results of this analysis revealed no similarity with any of the species analyzed, as all *p*-values were 0.001. Since this value does not exceed the threshold of 0.003 (3σ), all species showed statistically significant differences. In other words, the tooth marks from the Coímbre Cave do not correspond to any of the species included in the current database.

**Figure 7:**
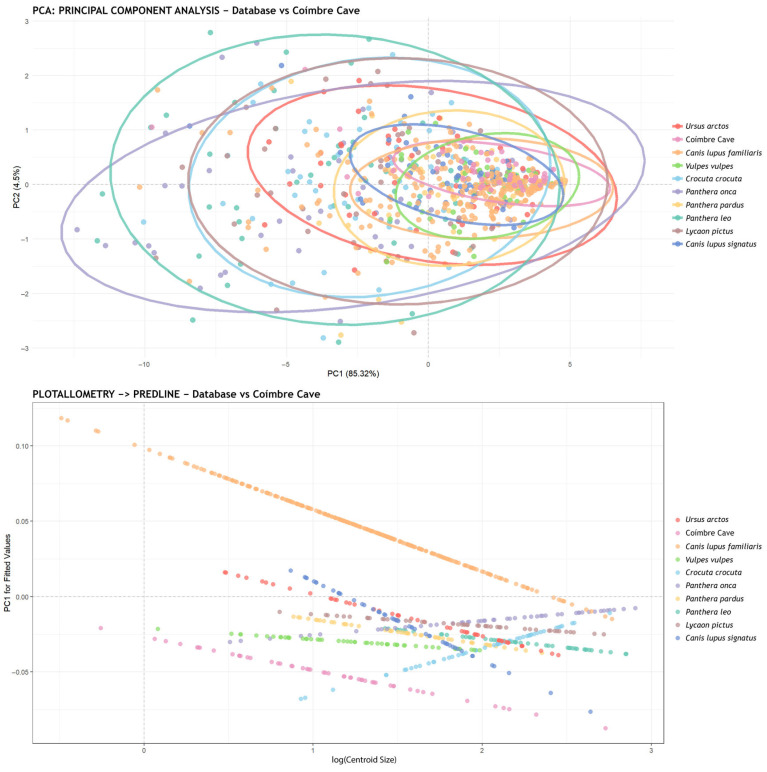
(**Top**) Principal component analysis (PCA) showing PC1 and PC2 values for the tooth marks from Coímbre Cave and all species included in the database. The data plotted were not scaled (scale = FALSE), meaning that both the size and shape of the marks were taken into account. (**Bottom**) Graph showing the allometric analysis using the PredLine method for all groups.

Figure 7 presents the PCA plot along with the corresponding concentration ellipses. This representation shows that the tooth marks from Coímbre Cave are significantly smaller in morphology compared to all other species—even smaller than those of *Canis lupus familiaris*, which are the smallest in the database. In the same figure, a graph of the allometric analysis using the “Predicted Lines” method is shown, illustrating the linear trends within the analyzed data.

### 4.2. Statistical Analysis: Humans

Considering the results obtained in previous analyses, an alternative line of investigation was pursued. The site in question corresponds to a human settlement where bones with signs of anthropogenic activity, such as cut marks, have been found. Based on the identification of significant differences in the previous values, it was decided to incorporate an experiment with tooth marks produced by modern humans into the database in order to determine whether humans could have been the taphonomic agent at this site—or to rule out that possibility.

In this context, 20 tooth pits produced by *Homo sapiens* were analyzed and compared with those from Coímbre Cave (a total of 728 pits: 50 from Coímbre, 658 from the previous database, and 20 from humans). To facilitate visualization of the results in the graphs, the data were grouped into four carnivore subfamilies and humans (*Caninae*, *Hyaeninae*, *Pantherinae*, *Ursinae*, and *Homo sapiens*), allowing for reduced overlap and data clustering—particularly of the concentration ellipses, which created visual noise.

First, a MANOVA was performed, revealing significant overall differences among the groups compared. Additionally, the Shapiro–Wilk test indicated that the values were not homogeneous. Consequently, a post-hoc analysis was conducted using Wilks’ lambda method, and the *p*-values obtained are presented in Table 2.

The results of this analysis show significant differences for all carnivore subfamilies, as none surpassed the threshold of 0.003 (3σ), except for *Homo sapiens*, whose *p*-value was 0.086 (Table 2). Therefore, the tooth marks from Coímbre Cave dating to the Late Magdalenian period are likely related to the human-produced marks from the experimental dataset.

**Figure 8:**
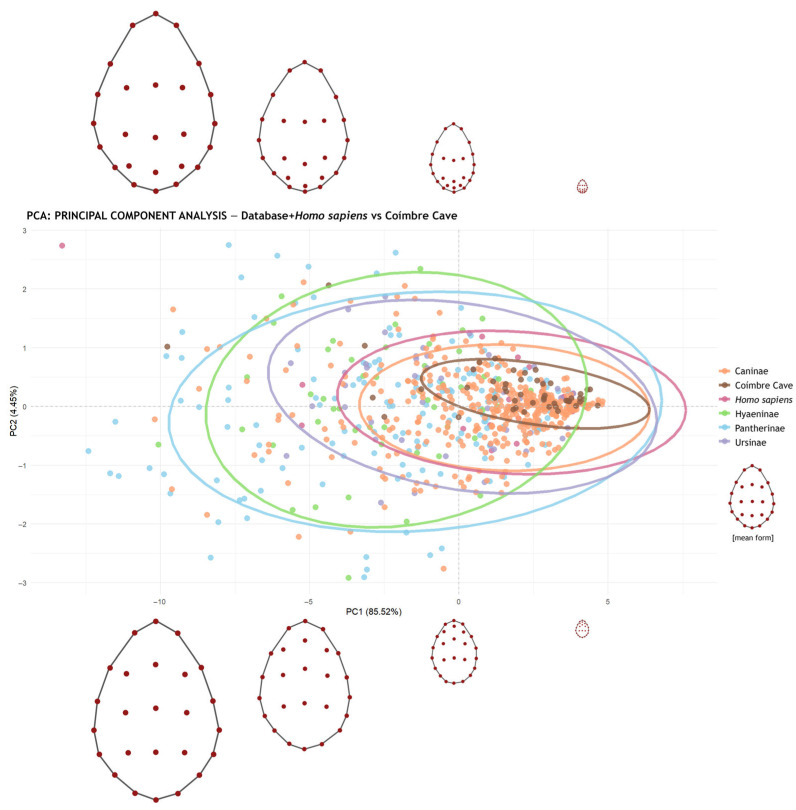
Principal component analysis (PCA) showing PC1 and PC2 values for the tooth marks from Coímbre Cave and the five subfamilies included in the database (including humans). The data plotted have not been scaled (scale = FALSE); in this case, both the size and shape of the marks have been taken into account. The figure also includes the distribution of landmarks and the varying sizes of the data. PC1 and PC2 together account for 89.71% of the total variability in the dataset (PC1 = 85.3%, PC2 = 4.41%).

Figure 8 shows the principal component analysis (PCA) plot, in which mark size was considered, along with the corresponding concentration ellipses. The PCA revealed that the first two components explain 89.71% of the total variance (PC1 = 85.52%, PC2 = 4.45%). As these two components capture most of the data variability, the subsequent analyses were based on them. Additionally, a distribution of the landmarks and the mark sizes is included, highlighting the notably small size of the tooth marks found on the bone surface of bone remains from Coímbre Cave.

## 5. Discussion

Taphonomic studies conducted on the Magdalenian levels of Coímbre Cave revealed that the faunal assemblage deposited at the site was heavily affected by human activity, showing numerous anthropogenic modifications such as cut marks, percussion marks, and evidence of thermal alteration [46,47,48]. However, the presence of certain tooth marks at the site raised the possibility that these may have been produced by canids [47]. Furthermore, the distribution and morphology of these marks suggested potential carnivore activity, prompting further investigation into the agents responsible for their formation. Canid bone remains have been identified at the site, suggesting that the presence of tooth marks produced by this group could be a plausible explanation. Moreover, the chronological context of the site (Upper Magdalenian) may have allowed for the presence of marks made by domestic dogs, as this is the time range in which researchers have proposed the existence of domesticated dogs, as previously observed at Erralla and Urtiaga [37,38,39].

However, as shown in Figure 8, the tooth marks on the bones from this site are very small and superficial, with concentration ellipses reflecting the positioning of the marks. Taphonomically, most of these marks are quite shallow on the cortical surface of the bones. This suggests that they are unlikely to be associated with large carnivores, such as wolves, which typically leave deeper marks due to their stronger bone-processing capabilities.

The analyses carried out indicate that the marks were likely produced by a human taphonomic agent. In addition, a significant portion of the bones show signs of cremation and a high number of cut marks [46,47,48], suggesting sustained and active human intervention in the processing of the bones. Therefore, these marks could represent the consumption of hunted prey, as well as the use of human teeth to remove remaining flesh from the consumed bones. This behavior could explain the more superficial morphology of many of the marks.

Nonetheless, deeper marks have also been identified, such as marks from furrowing, which involves the destruction of bone epiphyses and indicates more aggressive consumption—potentially linked to the activity of a carnivore. However, we have not yet been able to precisely determine the specific agent responsible. Still, some ethnographic studies have documented that humans can also intensively manipulate bones [77,78,79,80], even producing tooth marks that can be mistaken for those made by carnivores [81,82].

**Figure 9:**
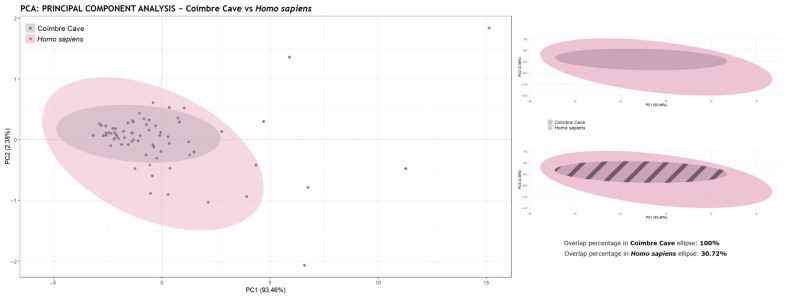
Principal component analysis (PCA) showing the PC1 and PC2 values for the tooth marks from Coímbre Cave (50 pits) and the experimental sample from modern *Homo sapiens* (20 pits). The data represented graphically were not scaled (scale = FALSE); in this case, both the size and shape of the marks were considered.

Figure 9 shows the difference between the concentration ellipses. When calculating the overlap of the ellipses, it was found that the ellipse for Coímbre is entirely overlapped with that of *Homo sapiens* (100%), indicating that the Coímbre marks are fully represented by the human tooth mark data—unlike the overlaps observed with *Vulpes vulpes*, *Canis lupus signatus*, and *Canis lupus familiaris* in Figure 6. All of this evidence is further supported by the post-hoc permutation MANOVA, which revealed no significant differences between the experimental human tooth marks and those from Coímbre, as well as by the results presented in Table 3, which compares the different Pearson correlation coefficient values.

These results are particularly interesting, as they allow us to document—archaeologically—a rarely recorded process: human tooth marks on ungulate bones. Although this phenomenon is not entirely new and has been reported in other contexts [51,83], the most noteworthy aspect of this analysis is the application of a novel technique that can help identify and differentiate human tooth marks from those produced by carnivores—a topic widely discussed in the literature [78,79,82,83,84,85]. However, despite these promising results, we believe it is essential to proceed with caution and carry out further experimental research to expand the sample of anthropogenic modifications on ungulates, as much of the existing research to date has focused on small mammals or birds [79,86,87,88].

## 6. Conclusions

The results of the analyses conducted in this study have been surprising, as we identified an unexpected finding relative to our working hypothesis. In examining the bone samples from Coímbre cave, our initial hypothesis proposed that the tooth marks on the bones could have been caused by canids, including domesticated dogs. However, our results suggest that these were not produced by canids or other carnivores but that their characteristics are consistently associated with human activity. This finding is significant, as it demonstrates how taphonomy, in combination with geometric morphometric analyses and robust statistical methods, constitutes an effective tool for identifying human tooth marks and distinguishing them from those produced by other carnivores.

Future research should aim to expand the experimental sample of human tooth marks in order to generate a broader range of marks with varying intensities. This would allow for the inclusion of both deeper and more superficial marks, across different bones and species, thereby contributing to a more comprehensive understanding of the alterations caused by human teeth on skeletal remains.

## Figures and Tables

**Table 1:** *p*-values from the post-hoc permutation test of the multivariate analysis of variance (MANOVA) for the tooth marks from Coímbre Cave and the various species in the database, using Wilks’ lambda method. The data plotted were not scaled (scale = FALSE), meaning that both the size and shape of the marks were taken into account.

	*Ursus arctos*	Coímbre Cave	*Canis lupus familiaris*	*Vulpes vulpes*	*Crocuta crocuta*	*Panthera onca*	*Panthera pardus*	*Panthera leo*	*Lycaon pictus*
Coímbre Cave	0.001	-	-	-	-	-	-	-	-
*Canis lupus familiaris*	0.001	0.001	-	-	-	-	-	-	-
*Vulpes vulpes*	0.001	0.001	0.001	-	-	-	-	-	-
*Crocuta crocuta*	0.001	0.001	0.001	0.001	-	-	-	-	-
*Panthera onca*	0.001	0.001	0.001	0.001	0.617	-	-	-	-
*Panthera pardus*	0.001	0.001	0.001	0.014	0.001	0.001	-	-	-
*Panthera leo*	0.001	0.001	0.001	0.001	0.415	0.748	0.001	-	-
*Lycaon pictus*	0.045	0.001	0.001	0.001	0.486	0.253	0.006	0.024	-
*Canis lupus signatus*	0.066	0.001	0.004	0.001	0.001	0.001	0.005	0.001	0.004

**Table 2:** *p*-values for the post-hoc permutation test of the multivariate analysis of variance (MANOVA) comparing tooth marks from Coímbre Cave with subfamilies and human samples, using Wilks’ lambda method. The data plotted have not been scaled (scale = FALSE); in this case, both the size and shape of the marks have been considered.

	Caninae	Coímbre Cave	*Homo sapiens*	Hyaeninae	Pantherinae
Coímbre Cave	0.001	-	-	-	-
*Homo sapiens*	0.362	**0.086**	-	-	-
Hyaeninae	0.001	0.001	0.001	-	-
Pantherinae	0.001	0.001	0.001	0.951	-
Ursinae	0.001	0.001	0.066	0.001	0.009

**Table 3:** Key comparisons between Coímbre and each of the studied species. The table presents *p*-values for the most relevant comparisons, allowing the identification of significant relationships and groups with substantial differences. Bold values indicate statistically significant differences (*p* < 0.003).

	Coímbre Cave
*Canis lupus signatus*	0.001
*Canis lupus familiaris*	0.001
*Vulpes vulpes*	0.001
*Lycaon pictus*	0.001
*Crocuta crocuta*	0.001
*Panthera onca*	0.001
*Panthera pardus*	0.001
*Panthera leo*	0.001
*Ursus arctos*	0.001
*Homo sapiens*	**0.086**

## Data Availability

The data are contained within Supplementary Materials, and the samples are in the University Complutense of Madrid.

## Supplementary Materials

The following supporting information can be downloaded at https://www.mdpi.com/article/10.3390/ani15091319/s1: Supplementary File S1. Reference framework for tooth marks produced by carnivores; Supplementary File S2. Data relating to Coímbre tooth marks.
