# Supplementary material for: Uncovering Human Tooth Marks in the Search for Dog Domestication: The Case of Coímbre Cave
Source: Animals (Basel). 2025 May 2;15(9):1319. doi: 10.3390/ani15091319 (PMC12070903; doi:10.3390/ani15091319)
Supplement: Supplementary file 1 [file animals-15-01319-s001.zip › animals-3605509-supplementary.pdf]

Supplementary Materials

Supplementary File S1

Canis lupus signatus (Iberian wolf): The marks come from captive individuals housed in the parks of Hosquillo (Cuenca, Spain) and Cabárceno (Cantabria, Spain), as well as from wild wolves in the parks of Villardeciervos and Flechas (Zamora, Spain). A total of 571 tooth marks were collected, found on goat, deer, and horse bones [61].

Canis lupus familiaris (domestic dog): The marks were produced by medium- and large-sized dog breeds, of both sexes and various individuals. The breeds were selected for their similarity to wolves in traits such as size and weight. These breeds included Labrador Retriever, Mastiff, Rottweiler, Irish Setter, Boxer, and a medium-sized mixed-breed dog [31].

Lycaon pictus (African wild dog): These marks were obtained from horse bones, such as radii and tibiae, during 2009 and 2010 in the Cabárceno park. They were produced by two individuals (one male and one female) [60].

Vulpes vulpes (red fox): The marks come from long sheep bones collected in the area of Ayllón (Segovia, Spain) in 2002 [67].

Ursus arctos (brown bear): The samples were collected in 2020 in the Cabárceno park, from several adult individuals of both sexes [33].

Crocuta crocuta (spotted hyena): These marks originated on tibiae and radii of horses provided to the Cabárceno park in 2012. All the marks were produced by a single adult female. Since hyenas tend to consume bones entirely, the samples used were only exposed to the animals for a few hours [33].

Panthera leo (lion): The marks were collected in 2011 in the Cabárceno park. The bones were horse radii and tibiae marked by individuals of both sexes [33].

Panthera onca (jaguar): These marks were obtained in 2009–2010 in the Cabárceno park, from horse tibiae and radii [68].

Panthera pardus (leopard): The marks were produced by individuals of both sexes of the subspecies P. p. kotiya (Sri Lankan leopard) housed in the Cabárceno park. The samples were collected in 2011 and correspond to horse bones [66].

Supplementary File S2

'Coimbre1

1.1719572e+001 1.3551652e+000 1.7866632e+001

1.2049899e+001 7.0346981e-001 1.7927294e+001

1.2638628e+001 3.3536994e-001 1.7897438e+001

1.3204800e+001 9.1148308e-004 1.7756878e+001

1.3839273e+001 -2.2217131e-001 1.7510593e+001

1.1520153e+001 2.1559541e+000 1.7679310e+001

1.1816715e+001 1.3071610e+000 1.7274422e+001

1.2506586e+001 6.5170914e-001 1.7051888e+001

1.3451770e+001 1.2907960e-001 1.6960272e+001

1.4654486e+001 -3.2851800e-001 1.6964048e+001

1.1673700e+001 2.6970053e+000 1.7375433e+001

1.1943585e+001 1.8638234e+000 1.6737036e+001

1.2632270e+001 1.1336378e+000 1.6397549e+001

1.3690372e+001 5.2947962e-001 1.6347179e+001

1.5040949e+001 -9.3234785e-002 1.6451580e+001

1.2059901e+001 2.9548206e+000 1.6964535e+001

1.2380023e+001 2.3583937e+000 1.6305206e+001

1.2962827e+001 1.7239699e+000 1.5895288e+001

1.3866947e+001 1.1293944e+000 1.5829403e+001

1.5000640e+001 4.8990005e-001 1.5967127e+001

1.2510276e+001 2.9883134e+000 1.6296488e+001

1.3018153e+001 2.7753003e+000 1.5845932e+001

1.3519783e+001 2.4450998e+000 1.5563478e+001

1.4062841e+001 2.0468094e+000 1.5541677e+001

1.4554323e+001 1.4576980e+000 1.5569265e+001

'Coimbre2

1.6996906e+001 -1.8721629e+000 1.6416878e+001

1.7902071e+001 -2.5779383e+000 1.6178392e+001

1.8966494e+001 -3.0353069e+000 1.5744546e+001

2.0145908e+001 -3.3075531e+000 1.5072214e+001

2.1450504e+001 -3.3816845e+000 1.4171395e+001

1.6182810e+001 -8.3832645e-001 1.6221308e+001

1.7224279e+001 -1.5036062e+000 1.5786302e+001

1.8373926e+001 -2.1727841e+000 1.4848291e+001

2.0197968e+001 -2.5590124e+000 1.3801072e+001

2.2977329e+001 -2.4624357e+000 1.3040430e+001

1.5842949e+001 2.0983132e-002 1.5747062e+001

1.6771666e+001 -6.1032331e-001 1.5046343e+001

1.8081688e+001 -1.1322045e+000 1.4021642e+001

2.0114624e+001 -1.4622763e+000 1.2760283e+001

2.3538334e+001 -1.3276364e+000 1.2530595e+001

1.6008789e+001 7.2781050e-001 1.5026047e+001

1.6866348e+001 4.2473319e-001 1.4254707e+001

1.8141180e+001 8.8131271e-002 1.3338182e+001

1.9985363e+001 -2.5054228e-001 1.2246999e+001

2.2910679e+001 -3.6584270e-001 1.1960997e+001

1.6599625e+001 1.2266936e+000 1.3976779e+001

1.7343662e+001 1.4875946e+000 1.3209471e+001

1.8310942e+001 1.4338170e+000 1.2494108e+001

1.9577276e+001 1.1038321e+000 1.1916563e+001

2.1169109e+001 5.5397797e-001 1.1561743e+001

'Coimbre3

2.0813126e+001 1.5262964e+000 2.2864748e+001

2.1063862e+001 1.2518249e+000 2.2759005e+001

2.1343761e+001 1.1255151e+000 2.2616001e+001

2.1650679e+001 1.1461179e+000 2.2435904e+001

2.1979292e+001 1.3038163e+000 2.2204002e+001

2.0646744e+001 1.8828477e+000 2.2875368e+001

2.0740124e+001 1.6590818e+000 2.2427200e+001

2.1010777e+001 1.5295053e+000 2.2117584e+001

2.1505121e+001 1.5108929e+000 2.1960632e+001

2.2215929e+001 1.6095229e+000 2.1949297e+001

2.0540514e+001 2.1946156e+000 2.2815453e+001

2.0624746e+001 2.0571713e+000 2.2238165e+001

2.0854103e+001 1.9446635e+000 2.1817589e+001

2.1447035e+001 1.9294676e+000 2.1740105e+001

2.2287374e+001 1.9450209e+000 2.1826429e+001

2.0637056e+001 2.4747365e+000 2.2718004e+001

2.0670761e+001 2.4818749e+000 2.2172276e+001

2.0921749e+001 2.4512119e+000 2.1791637e+001

2.1472088e+001 2.3909733e+000 2.1762913e+001

2.2178469e+001 2.2906926e+000 2.1787086e+001

2.0792009e+001 2.7346635e+000 2.2541466e+001

2.1030289e+001 2.9454718e+000 2.2343817e+001

2.1262001e+001 3.0231917e+000 2.2118246e+001

2.1550341e+001 2.9269903e+000 2.1938231e+001

2.1890724e+001 2.6514614e+000 2.1829062e+001

'Coimbre4

2.6558062e+001 -2.8739445e+000 1.9897757e+001

2.6656609e+001 -2.9484820e+000 1.9804562e+001

2.6769163e+001 -2.9781606e+000 1.9725681e+001

2.6896894e+001 -2.9613686e+000 1.9666096e+001

2.7034910e+001 -2.9037914e+000 1.9609461e+001

2.6496761e+001 -2.7284877e+000 1.9933979e+001

2.6570547e+001 -2.7308359e+000 1.9757347e+001

2.6733345e+001 -2.7145827e+000 1.9682446e+001

2.6928280e+001 -2.7176406e+000 1.9642620e+001

2.7168272e+001 -2.7388563e+000 1.9546623e+001

2.6471001e+001 -2.5889077e+000 1.9936691e+001

2.6550631e+001 -2.5395687e+000 1.9742321e+001

2.6679050e+001 -2.5280504e+000 1.9595016e+001

2.6925371e+001 -2.5344276e+000 1.9589556e+001

2.7217775e+001 -2.5816040e+000 1.9547623e+001

2.6474688e+001 -2.4495094e+000 1.9903267e+001

2.6574642e+001 -2.3800650e+000 1.9743885e+001

2.6721775e+001 -2.3545289e+000 1.9652336e+001

2.6913088e+001 -2.3733764e+000 1.9581556e+001

2.7175608e+001 -2.4352744e+000 1.9561705e+001

2.6555208e+001 -2.3192618e+000 1.9849274e+001

2.6655725e+001 -2.2352836e+000 1.9766237e+001

2.6775774e+001 -2.2086768e+000 1.9705700e+001

2.6906420e+001 -2.2333376e+000 1.9661329e+001

2.7045328e+001 -2.3004589e+000 1.9612263e+001

'Coimbre5

2.1313021e+001 3.5463820e+000 1.4977585e+001

2.1362165e+001 3.2301049e+000 1.4918712e+001

2.1470551e+001 2.9745643e+000 1.4807388e+001

2.1661013e+001 2.7912576e+000 1.4657250e+001

2.1900375e+001 2.6581929e+000 1.4441628e+001

2.1358665e+001 3.8853896e+000 1.4914799e+001

2.1358234e+001 3.5975478e+000 1.4765363e+001

2.1456753e+001 3.2774491e+000 1.4555190e+001

2.1783768e+001 2.9453652e+000 1.4357899e+001

2.2213787e+001 2.5705993e+000 1.4099441e+001

2.1463928e+001 4.1365490e+000 1.4801786e+001

2.1451426e+001 3.8987174e+000 1.4571517e+001

2.1595558e+001 3.5741973e+000 1.4330428e+001

2.1935926e+001 3.1660850e+000 1.4109334e+001

2.2378475e+001 2.6583457e+000 1.3828087e+001

2.1686871e+001 4.2610016e+000 1.4668768e+001

2.1729731e+001 4.1198797e+000 1.4380490e+001

2.1941187e+001 3.8447127e+000 1.4176975e+001

2.2142979e+001 3.4655969e+000 1.3934427e+001

2.2465702e+001 2.9540606e+000 1.3725211e+001

2.1906631e+001 4.3526807e+000 1.4443582e+001

2.2084427e+001 4.2952862e+000 1.4129325e+001

2.2227779e+001 4.1263733e+000 1.3903677e+001

2.2337391e+001 3.8422332e+000 1.3764346e+001

2.2440344e+001 3.4441364e+000 1.3741617e+001

'Coimbre6

1.4493326e+001 1.3627018e+000 1.2811611e+001

1.4648774e+001 1.9137565e+000 1.2571665e+001

1.4620819e+001 2.5399621e+000 1.2401277e+001

1.4384210e+001 3.2269588e+000 1.2258307e+001

1.3949474e+001 3.9811032e+000 1.2159731e+001

1.4083148e+001 9.0351778e-001 1.3207700e+001

1.4150281e+001 1.3157493e+000 1.3029541e+001

1.3996615e+001 2.1201024e+000 1.2822516e+001

1.3595193e+001 3.2664499e+000 1.2577914e+001

1.2977627e+001 4.7496295e+000 1.2321651e+001

1.3622328e+001 7.3340505e-001 1.3590240e+001

1.3509961e+001 1.0679089e+000 1.3451312e+001

1.3159079e+001 1.8379705e+000 1.3161265e+001

1.2903100e+001 3.2482364e+000 1.3016555e+001

1.2324072e+001 4.9697304e+000 1.2650546e+001

1.3100422e+001 8.4833050e-001 1.3947084e+001

1.2890401e+001 1.2530171e+000 1.4040791e+001

1.2496000e+001 1.9710802e+000 1.3817506e+001

1.2381904e+001 3.2123587e+000 1.3676019e+001

1.2070587e+001 4.7230077e+000 1.3234571e+001

1.2541723e+001 1.2579368e+000 1.4305646e+001

1.2050953e+001 1.7916930e+000 1.4577024e+001

1.1821474e+001 2.4016275e+000 1.4614570e+001

1.1832972e+001 3.0843728e+000 1.4395752e+001

1.2026320e+001 3.8252745e+000 1.3872165e+001

'Coimbre7

1.1289712e+001 -3.4440420e+000 1.5045982e+001

1.1362970e+001 -3.7026060e+000 1.4661126e+001

1.1481456e+001 -3.8366294e+000 1.4291217e+001

1.1653546e+001 -3.8485391e+000 1.3936993e+001

1.1912454e+001 -3.7425663e+000 1.3615791e+001

1.1269338e+001 -3.0504026e+000 1.5399966e+001

1.1364013e+001 -3.1824150e+000 1.4949188e+001

1.1439059e+001 -3.2564230e+000 1.4407720e+001

1.1754960e+001 -3.3619170e+000 1.3865048e+001

1.2255271e+001 -3.4797823e+000 1.3306613e+001

1.1363374e+001 -2.6768422e+000 1.5547801e+001

1.1494286e+001 -2.7166002e+000 1.5096460e+001

1.1528103e+001 -2.7142887e+000 1.4473103e+001

1.1888145e+001 -2.8631110e+000 1.3850736e+001

1.2471739e+001 -3.1181004e+000 1.3202737e+001

1.1506454e+001 -2.2984705e+000 1.5477456e+001

1.1640739e+001 -2.2264769e+000 1.5045725e+001

1.1827257e+001 -2.2464900e+000 1.4536047e+001

1.2141046e+001 -2.3909712e+000 1.3959010e+001

1.2582552e+001 -2.6658823e+000 1.3321752e+001

1.1756540e+001 -1.9385211e+000 1.5198594e+001

1.1993919e+001 -1.7980618e+000 1.4874846e+001

1.2207948e+001 -1.7799406e+000 1.4509145e+001

1.2374641e+001 -1.8726479e+000 1.4085524e+001

1.2588942e+001 -2.1238484e+000 1.3663525e+001

'Coimbre8

1.8029587e+001 -4.7466812e+000 2.2233349e+001

1.8174667e+001 -5.0424972e+000 2.2075825e+001

1.8416437e+001 -5.2433939e+000 2.1973768e+001

1.8642519e+001 -5.3403678e+000 2.1791918e+001

1.8900448e+001 -5.3396020e+000 2.1591627e+001

1.7993538e+001 -4.4209900e+000 2.2230337e+001

1.8149723e+001 -4.6074662e+000 2.2128479e+001

1.8407351e+001 -4.8040199e+000 2.1913986e+001

1.8827854e+001 -5.0073838e+000 2.1669508e+001

1.9320822e+001 -5.2063303e+000 2.1286451e+001

1.8019758e+001 -4.1430898e+000 2.2147242e+001

1.8149893e+001 -4.2351389e+000 2.2015116e+001

1.8399462e+001 -4.4115329e+000 2.1752258e+001

1.8901270e+001 -4.6497231e+000 2.1500111e+001

1.9534740e+001 -4.9679117e+000 2.1145206e+001

1.8136478e+001 -3.9151890e+000 2.2034838e+001

1.8266052e+001 -3.9285226e+000 2.1831017e+001

1.8540443e+001 -4.0528922e+000 2.1619465e+001

1.8965248e+001 -4.2800531e+000 2.1395199e+001

1.9532911e+001 -4.6167173e+000 2.1155180e+001

1.8310833e+001 -3.7293468e+000 2.1828329e+001

1.8549118e+001 -3.6718748e+000 2.1627991e+001

1.8804754e+001 -3.7263746e+000 2.1498758e+001

1.9042088e+001 -3.8867111e+000 2.1370550e+001

1.9265451e+001 -4.1574526e+000 2.1248974e+001

'Coimbre9

2.3375967e+001 -2.7828543e+000 1.9748030e+001

2.3385748e+001 -2.6891918e+000 1.9756792e+001

2.3372717e+001 -2.6089554e+000 1.9775175e+001

2.3352627e+001 -2.5448692e+000 1.9829235e+001

2.3327984e+001 -2.4999318e+000 1.9920094e+001

2.3378965e+001 -2.8824389e+000 1.9780903e+001

2.3353905e+001 -2.8488104e+000 1.9790831e+001

2.3311039e+001 -2.7730412e+000 1.9801172e+001

2.3273001e+001 -2.6504040e+000 1.9866663e+001

2.3289799e+001 -2.4910429e+000 2.0022205e+001

2.3364483e+001 -2.9477866e+000 1.9836222e+001

2.3324753e+001 -2.9372787e+000 1.9844692e+001

2.3251631e+001 -2.8685069e+000 1.9847992e+001

2.3215136e+001 -2.7283885e+000 1.9931477e+001

2.3268984e+001 -2.5223401e+000 2.0100357e+001

2.3305941e+001 -2.9895344e+000 1.9885168e+001

2.3274256e+001 -2.9661846e+000 1.9915886e+001

2.3222803e+001 -2.9015810e+000 1.9952896e+001

2.3187378e+001 -2.7766628e+000 2.0025978e+001

2.3250910e+001 -2.5913699e+000 2.0147919e+001

2.3267281e+001 -2.9781442e+000 1.9986557e+001

2.3253799e+001 -2.9278007e+000 2.0055548e+001

2.3237871e+001 -2.8667550e+000 2.0109255e+001

2.3237061e+001 -2.7894843e+000 2.0145504e+001

2.3233875e+001 -2.6987400e+000 2.0165529e+001

'Coimbre10

1.4903986e+001 2.0567076e+000 2.1197735e+001

1.4721164e+001 2.1070855e+000 2.1418020e+001

1.4565295e+001 2.0782497e+000 2.1651581e+001

1.4404373e+001 1.9707396e+000 2.1873499e+001

1.4280642e+001 1.7786682e+000 2.2115995e+001

1.5066744e+001 1.8559506e+000 2.0954292e+001

1.4903709e+001 2.0011899e+000 2.1192724e+001

1.4652314e+001 1.9655242e+000 2.1475178e+001

1.4379733e+001 1.7678788e+000 2.1863646e+001

1.4134728e+001 1.3892968e+000 2.2387802e+001

1.5178888e+001 1.6604815e+000 2.0876120e+001

1.4994739e+001 1.7955220e+000 2.1076660e+001

1.4708252e+001 1.7483648e+000 2.1363300e+001

1.4421492e+001 1.5262219e+000 2.1826702e+001

1.4094223e+001 1.1279529e+000 2.2432335e+001

1.5138703e+001 1.4479421e+000 2.0868931e+001

1.4941810e+001 1.4832999e+000 2.1015432e+001

1.4736644e+001 1.4196910e+000 2.1314777e+001

1.4483438e+001 1.2516594e+000 2.1728691e+001

1.4189483e+001 9.7906363e-001 2.2263954e+001

1.5076914e+001 1.2343215e+000 2.1059134e+001

1.4962510e+001 1.0866473e+000 2.1211695e+001

1.4825503e+001 9.8943418e-001 2.1409058e+001

1.4656794e+001 9.4129407e-001 2.1642315e+001

1.4457471e+001 9.4180161e-001 2.1912289e+001

'Coimbre11

1.6358110e+001 -1.5818133e+000 1.6663908e+001

1.6362741e+001 -1.5969765e+000 1.6523211e+001

1.6417589e+001 -1.5872273e+000 1.6403421e+001

1.6507212e+001 -1.5514948e+000 1.6306494e+001

1.6592707e+001 -1.4924585e+000 1.6191862e+001

1.6306894e+001 -1.5051059e+000 1.6751047e+001

1.6355160e+001 -1.5674613e+000 1.6599800e+001

1.6398840e+001 -1.5505700e+000 1.6425241e+001

1.6525999e+001 -1.4781020e+000 1.6252930e+001

1.6759029e+001 -1.3592205e+000 1.6116861e+001

1.6313656e+001 -1.4127777e+000 1.6809561e+001

1.6347166e+001 -1.4693619e+000 1.6648439e+001

1.6396381e+001 -1.4363884e+000 1.6461700e+001

1.6549667e+001 -1.3513442e+000 1.6269911e+001

1.6815742e+001 -1.2263249e+000 1.6092833e+001

1.6321867e+001 -1.2997030e+000 1.6813480e+001

1.6354927e+001 -1.2927910e+000 1.6678240e+001

1.6441341e+001 -1.2607788e+000 1.6524475e+001

1.6550800e+001 -1.1762998e+000 1.6332045e+001

1.6779972e+001 -1.0949181e+000 1.6148252e+001

1.6357796e+001 -1.1687678e+000 1.6774872e+001

1.6388556e+001 -1.0492613e+000 1.6691250e+001

1.6438709e+001 -9.7474974e-001 1.6577881e+001

1.6519264e+001 -9.4476885e-001 1.6432760e+001

1.6648350e+001 -9.6626532e-001 1.6279289e+001

'Coimbre12

1.5403586e+001 9.2598587e-001 1.3797904e+001

1.5456735e+001 8.0659205e-001 1.3689887e+001

1.5541348e+001 7.1722496e-001 1.3567814e+001

1.5673754e+001 6.6904157e-001 1.3434789e+001

1.5836737e+001 6.4971679e-001 1.3282331e+001

1.5408436e+001 1.1218191e+000 1.3852206e+001

1.5383053e+001 1.0225732e+000 1.3625257e+001

1.5518278e+001 9.3547672e-001 1.3433676e+001

1.5752150e+001 8.5021269e-001 1.3271580e+001

1.6039490e+001 7.4687296e-001 1.3096415e+001

1.5452567e+001 1.2752670e+000 1.3863699e+001

1.5509794e+001 1.2162544e+000 1.3629564e+001

1.5557499e+001 1.1491054e+000 1.3376838e+001

1.5810064e+001 1.0398214e+000 1.3194157e+001

1.6147165e+001 8.9865094e-001 1.3018108e+001

1.5536209e+001 1.3837167e+000 1.3834205e+001

1.5637945e+001 1.3633064e+000 1.3647573e+001

1.5692148e+001 1.3558443e+000 1.3414639e+001

1.5871471e+001 1.2644252e+000 1.3215489e+001

1.6179806e+001 1.0961411e+000 1.3069753e+001

1.5648139e+001 1.4576576e+000 1.3757632e+001

1.5772255e+001 1.5373627e+000 1.3655266e+001

1.5857730e+001 1.5758892e+000 1.3510036e+001

1.5968867e+001 1.5145308e+000 1.3359427e+001

1.6090393e+001 1.3663075e+000 1.3196838e+001

'Coimbre13

1.7777487e+001 -3.9488082e+000 1.5557301e+001

1.7878881e+001 -4.0970302e+000 1.5499688e+001

1.8039940e+001 -4.1922288e+000 1.5424812e+001

1.8239399e+001 -4.2416687e+000 1.5305076e+001

1.8475393e+001 -4.2464485e+000 1.5137063e+001

1.7693968e+001 -3.7566009e+000 1.5581354e+001

1.7828667e+001 -3.9020917e+000 1.5505050e+001

1.8018599e+001 -4.0186024e+000 1.5358065e+001

1.8302645e+001 -4.0994282e+000 1.5172873e+001

1.8696136e+001 -4.1423731e+000 1.4965056e+001

1.7666546e+001 -3.5781806e+000 1.5553630e+001

1.7815105e+001 -3.6908531e+000 1.5452006e+001

1.8024372e+001 -3.7992570e+000 1.5286343e+001

1.8325161e+001 -3.8986444e+000 1.5077675e+001

1.8775663e+001 -3.9839182e+000 1.4879014e+001

1.7705650e+001 -3.4102890e+000 1.5486258e+001

1.7847670e+001 -3.4573674e+000 1.5358598e+001

1.8055031e+001 -3.5342655e+000 1.5207498e+001

1.8334068e+001 -3.6402080e+000 1.5035979e+001

1.8713314e+001 -3.7712874e+000 1.4878078e+001

1.7814184e+001 -3.2521958e+000 1.5382113e+001

1.7961176e+001 -3.1986237e+000 1.5247925e+001

1.8130198e+001 -3.2216058e+000 1.5138706e+001

1.8313974e+001 -3.3231864e+000 1.5045874e+001

1.8508795e+001 -3.5045373e+000 1.4961712e+001

'Coimbre14

2.1425377e+001 -4.8190746e+000 1.3273517e+001

2.1449421e+001 -5.0136909e+000 1.3234580e+001

2.1501087e+001 -5.1681080e+000 1.3139450e+001

2.1571318e+001 -5.2849531e+000 1.2986177e+001

2.1674480e+001 -5.3581004e+000 1.2779517e+001

2.1430065e+001 -4.5821886e+000 1.3252913e+001

2.1415907e+001 -4.7174902e+000 1.3155516e+001

2.1349110e+001 -4.8824158e+000 1.3013724e+001

2.1467575e+001 -5.0966768e+000 1.2800424e+001

2.1812418e+001 -5.3395381e+000 1.2552409e+001

2.1444048e+001 -4.4162383e+000 1.3169647e+001

2.1417509e+001 -4.5205808e+000 1.3042530e+001

2.1351297e+001 -4.6659956e+000 1.2873788e+001

2.1540445e+001 -4.9138036e+000 1.2659199e+001

2.1893837e+001 -5.2442760e+000 1.2401965e+001

2.1517162e+001 -4.3266916e+000 1.3031759e+001

2.1519011e+001 -4.4009180e+000 1.2899955e+001

2.1510771e+001 -4.5221796e+000 1.2722915e+001

2.1624966e+001 -4.7439055e+000 1.2519560e+001

2.1931786e+001 -5.0701666e+000 1.2340250e+001

2.1626127e+001 -4.3095398e+000 1.2834333e+001

2.1712831e+001 -4.3406482e+000 1.2677541e+001

2.1797852e+001 -4.4410834e+000 1.2552053e+001

2.1844007e+001 -4.5971851e+000 1.2421583e+001

2.1904375e+001 -4.8188958e+000 1.2345764e+001

'Coimbre15

1.7525557e+001 -3.0157995e+000 1.1572989e+001

1.7808043e+001 -3.1852748e+000 1.1439386e+001

1.8109663e+001 -3.3146210e+000 1.1248875e+001

1.8416000e+001 -3.4413209e+000 1.1005384e+001

1.8730417e+001 -3.5563829e+000 1.0707380e+001

1.7246714e+001 -2.8108051e+000 1.1427677e+001

1.7275063e+001 -3.1164553e+000 1.1281769e+001

1.7621675e+001 -3.3424325e+000 1.1033909e+001

1.8243706e+001 -3.5202453e+000 1.0693285e+001

1.9058275e+001 -3.7519794e+000 1.0190472e+001

1.7090858e+001 -2.6440132e+000 1.1207400e+001

1.7057802e+001 -2.9465215e+000 1.1025157e+001

1.7339104e+001 -3.2323833e+000 1.0747309e+001

1.8088671e+001 -3.4308968e+000 1.0379140e+001

1.9185871e+001 -3.6078072e+000 9.8917131e+000

1.7058910e+001 -2.5161567e+000 1.0913934e+001

1.7096889e+001 -2.7008219e+000 1.0690595e+001

1.7198484e+001 -2.9905858e+000 1.0359186e+001

1.7830530e+001 -3.2202787e+000 1.0003969e+001

1.8936335e+001 -3.3366702e+000 9.6801062e+000

1.7130375e+001 -2.4393802e+000 1.0547154e+001

1.7274063e+001 -2.4188433e+000 1.0236879e+001

1.7509766e+001 -2.4990802e+000 9.9641294e+000

1.7687843e+001 -2.8210928e+000 9.6497993e+000

1.8338867e+001 -2.9044898e+000 9.5774250e+000

'Coimbre16

1.9973310e+001 -3.8902843e+000 1.1131332e+001

2.0010790e+001 -4.0543995e+000 1.1119627e+001

2.0054230e+001 -4.2413874e+000 1.1026793e+001

2.0223635e+001 -4.3720160e+000 1.0952879e+001

2.0434813e+001 -4.5220776e+000 1.0831683e+001

1.9982811e+001 -3.6514730e+000 1.1084020e+001

2.0004967e+001 -3.9628952e+000 1.1126295e+001

2.0008018e+001 -4.1699286e+000 1.0814246e+001

2.0287910e+001 -4.3672218e+000 1.0744957e+001

2.0790178e+001 -4.4925861e+000 1.0805910e+001

2.0071535e+001 -3.4975836e+000 1.1039429e+001

2.0045179e+001 -3.8991730e+000 1.1078744e+001

2.0091583e+001 -4.0433431e+000 1.0636023e+001

2.0422911e+001 -4.2643495e+000 1.0588901e+001

2.0992456e+001 -4.4262633e+000 1.0736403e+001

2.0232697e+001 -3.4278991e+000 1.0983463e+001

2.0220036e+001 -3.6243806e+000 1.0727534e+001

2.0360151e+001 -3.8331437e+000 1.0588692e+001

2.0643396e+001 -4.0461903e+000 1.0568901e+001

2.1075096e+001 -4.2559862e+000 1.0679863e+001

2.0451675e+001 -3.4185781e+000 1.0892183e+001

2.0646927e+001 -3.4568439e+000 1.0808309e+001

2.0799116e+001 -3.5558486e+000 1.0690623e+001

2.0938963e+001 -3.7430429e+000 1.0685309e+001

2.1035883e+001 -3.9987481e+000 1.0632734e+001

'Coimbre17

3.0420929e+001 8.3106937e+000 1.4759333e+001

3.0365246e+001 8.4075718e+000 1.4667115e+001

3.0285561e+001 8.5029240e+000 1.4612086e+001

3.0178045e+001 8.5891256e+000 1.4585266e+001

3.0050997e+001 8.6809187e+000 1.4606278e+001

3.0424631e+001 8.2311869e+000 1.4862664e+001

3.0317131e+001 8.3258753e+000 1.4785151e+001

3.0180492e+001 8.4164019e+000 1.4697499e+001

3.0023514e+001 8.5573015e+000 1.4666131e+001

2.9818413e+001 8.7333851e+000 1.4683492e+001

3.0373343e+001 8.1635418e+000 1.4936622e+001

3.0231281e+001 8.2340269e+000 1.4868699e+001

3.0070082e+001 8.3061628e+000 1.4748468e+001

2.9887333e+001 8.4792252e+000 1.4738297e+001

2.9687452e+001 8.7304792e+000 1.4798802e+001

3.0276953e+001 8.1349354e+000 1.5010984e+001

3.0124760e+001 8.2011423e+000 1.4993684e+001

2.9964317e+001 8.2950726e+000 1.4957387e+001

2.9796875e+001 8.4315796e+000 1.4916820e+001

2.9633265e+001 8.6458578e+000 1.4921512e+001

3.0143293e+001 8.1654615e+000 1.5105975e+001

2.9972059e+001 8.2090797e+000 1.5167183e+001

2.9842348e+001 8.2876158e+000 1.5187312e+001

2.9747961e+001 8.3881769e+000 1.5156065e+001

2.9668303e+001 8.4924707e+000 1.5064297e+001

'Coimbre18

2.8552420e+001 4.7313080e+000 1.8713081e+001

2.8497499e+001 4.8949909e+000 1.8659485e+001

2.8402502e+001 5.0360355e+000 1.8646822e+001

2.8284565e+001 5.1661468e+000 1.8700609e+001

2.8105776e+001 5.2587066e+000 1.8761189e+001

2.8578146e+001 4.5822062e+000 1.8817804e+001

2.8470570e+001 4.7931895e+000 1.8732975e+001

2.8302935e+001 4.9633679e+000 1.8699768e+001

2.8124777e+001 5.1287789e+000 1.8797709e+001

2.7865566e+001 5.2499299e+000 1.8933523e+001

2.8543217e+001 4.4681630e+000 1.8922970e+001

2.8385031e+001 4.6595879e+000 1.8825081e+001

2.8181551e+001 4.8276320e+000 1.8791965e+001

2.7992916e+001 5.0103898e+000 1.8913012e+001

2.7737015e+001 5.1650896e+000 1.9085859e+001

2.8432369e+001 4.3733559e+000 1.9000544e+001

2.8275503e+001 4.5170555e+000 1.8992048e+001

2.8081753e+001 4.6532946e+000 1.8994589e+001

2.7893356e+001 4.8143859e+000 1.9067209e+001

2.7703119e+001 4.9945970e+000 1.9199137e+001

2.8291105e+001 4.3449950e+000 1.9134159e+001

2.8139606e+001 4.3653655e+000 1.9242668e+001

2.8020500e+001 4.4503441e+000 1.9318026e+001

2.7902216e+001 4.5785980e+000 1.9335464e+001

2.7783001e+001 4.7491264e+000 1.9293184e+001

'Coimbre19

2.8611120e+001 5.0451750e-001 2.0372355e+001

2.8757673e+001 4.7955883e-001 2.0308283e+001

2.8855932e+001 4.8574758e-001 2.0189154e+001

2.8974949e+001 5.6479263e-001 2.0085291e+001

2.9066265e+001 6.8648511e-001 1.9949856e+001

2.8464725e+001 6.2929797e-001 2.0430351e+001

2.8600725e+001 6.4790446e-001 2.0223219e+001

2.8752539e+001 6.9831169e-001 2.0059717e+001

2.8935415e+001 7.8318304e-001 1.9942120e+001

2.9121904e+001 8.7949121e-001 1.9794508e+001

2.8377346e+001 7.7468675e-001 2.0449656e+001

2.8449057e+001 8.0637068e-001 2.0145254e+001

2.8618000e+001 8.7814546e-001 1.9953745e+001

2.8849539e+001 9.7264826e-001 1.9867077e+001

2.9108580e+001 1.0627713e+000 1.9767195e+001

2.8331900e+001 9.3293971e-001 2.0418852e+001

2.8369690e+001 1.0139349e+000 2.0151323e+001

2.8536640e+001 1.0983672e+000 1.9996782e+001

2.8741104e+001 1.1629651e+000 1.9881397e+001

2.8997456e+001 1.2139342e+000 1.9799456e+001

2.8344009e+001 1.1088994e+000 2.0346701e+001

2.8402786e+001 1.2490017e+000 2.0264580e+001

2.8488274e+001 1.3361717e+000 2.0157993e+001

2.8628187e+001 1.3637105e+000 2.0048466e+001

2.8797796e+001 1.3405269e+000 1.9913223e+001

'Coimbre20

1.8373875e+001 3.3767228e+000 2.2964180e+001

1.8278723e+001 3.6798453e+000 2.2857794e+001

1.8110746e+001 3.9374716e+000 2.2850689e+001

1.7868206e+001 4.1473856e+000 2.2939411e+001

1.7548336e+001 4.3087783e+000 2.3120275e+001

1.8451818e+001 3.0722079e+000 2.3116190e+001

1.8196033e+001 3.2187564e+000 2.3166018e+001

1.7894154e+001 3.4751449e+000 2.3208410e+001

1.7565199e+001 3.8566129e+000 2.3280655e+001

1.7230274e+001 4.3704190e+000 2.3402956e+001

1.8399967e+001 2.8567555e+000 2.3299278e+001

1.8081045e+001 2.9246726e+000 2.3464437e+001

1.7734562e+001 3.1704383e+000 2.3557644e+001

1.7403019e+001 3.6299355e+000 2.3636095e+001

1.7037163e+001 4.2666836e+000 2.3636358e+001

1.8237230e+001 2.7511609e+000 2.3538404e+001

1.7950142e+001 2.8134570e+000 2.3768543e+001

1.7634628e+001 3.0279422e+000 2.3881552e+001

1.7297977e+001 3.4142299e+000 2.3889580e+001

1.6976854e+001 3.9985778e+000 2.3831335e+001

1.7961491e+001 2.7534044e+000 2.3829876e+001

1.7724035e+001 2.8096681e+000 2.4002218e+001

1.7505657e+001 2.9755149e+000 2.4096012e+001

1.7287830e+001 3.2333148e+000 2.4098143e+001

1.7084402e+001 3.5947902e+000 2.4017593e+001

'Coimbre21

1.7997066e+001 -9.4160360e-001 2.2866421e+001

1.7853708e+001 -8.6473250e-001 2.2929426e+001

1.7749701e+001 -8.4591252e-001 2.3028189e+001

1.7668570e+001 -8.8464928e-001 2.3147541e+001

1.7620064e+001 -9.8072600e-001 2.3295647e+001

1.8112263e+001 -1.0671983e+000 2.2841860e+001

1.7953604e+001 -1.0492393e+000 2.2921618e+001

1.7812996e+001 -1.0571495e+000 2.3055885e+001

1.7696966e+001 -1.0820745e+000 2.3250820e+001

1.7555054e+001 -1.1382239e+000 2.3468596e+001

1.8183014e+001 -1.1933087e+000 2.2877165e+001

1.8040346e+001 -1.2178303e+000 2.2978804e+001

1.7862085e+001 -1.2493049e+000 2.3086535e+001

1.7701725e+001 -1.2692165e+000 2.3279968e+001

1.7570253e+001 -1.2759632e+000 2.3561632e+001

1.8181255e+001 -1.3352354e+000 2.2935362e+001

1.8075800e+001 -1.3875350e+000 2.3042141e+001

1.7934343e+001 -1.4249797e+000 2.3162098e+001

1.7805275e+001 -1.4234471e+000 2.3350447e+001

1.7646646e+001 -1.4026362e+000 2.3561859e+001

1.8133524e+001 -1.4767015e+000 2.3046259e+001

1.8100420e+001 -1.5474665e+000 2.3174641e+001

1.8038261e+001 -1.5738612e+000 2.3293074e+001

1.7933805e+001 -1.5643952e+000 2.3391394e+001

1.7799562e+001 -1.5104601e+000 2.3479383e+001

'Coimbre22

1.8119493e+001 -1.4144762e+000 2.4372772e+001

1.8053051e+001 -1.5409296e+000 2.4491119e+001

1.8063578e+001 -1.6626414e+000 2.4588699e+001

1.8096090e+001 -1.8101737e+000 2.4619724e+001

1.8210138e+001 -1.9361221e+000 2.4638317e+001

1.8218557e+001 -1.3405330e+000 2.4266848e+001

1.8198689e+001 -1.5007387e+000 2.4339329e+001

1.8236036e+001 -1.6645389e+000 2.4434511e+001

1.8304653e+001 -1.8458030e+000 2.4533625e+001

1.8383560e+001 -2.0722013e+000 2.4607050e+001

1.8309834e+001 -1.3285670e+000 2.4176687e+001

1.8347971e+001 -1.4855877e+000 2.4230083e+001

1.8381123e+001 -1.6811297e+000 2.4295397e+001

1.8474712e+001 -1.8557886e+000 2.4440376e+001

1.8517632e+001 -2.1191823e+000 2.4539131e+001

1.8405743e+001 -1.3742731e+000 2.4117678e+001

1.8492096e+001 -1.5055457e+000 2.4154140e+001

1.8549753e+001 -1.6704313e+000 2.4215942e+001

1.8602596e+001 -1.8476400e+000 2.4327917e+001

1.8617804e+001 -2.0701945e+000 2.4444366e+001

1.8515018e+001 -1.4682192e+000 2.4096460e+001

1.8621248e+001 -1.5749192e+000 2.4101978e+001

1.8691406e+001 -1.6799997e+000 2.4146620e+001

1.8708729e+001 -1.8005493e+000 2.4215408e+001

1.8677979e+001 -1.9316841e+000 2.4312201e+001

'Coimbre23

9.3015270e+000 3.5754039e+000 9.9159002e+000

9.4371929e+000 3.3580220e+000 9.7851067e+000

9.6516876e+000 3.2367499e+000 9.7260494e+000

9.9639931e+000 3.2066219e+000 9.6446104e+000

1.0283226e+001 3.1488392e+000 9.4262171e+000

9.2838678e+000 3.8362892e+000 9.9961796e+000

9.4402990e+000 3.6182539e+000 9.8444643e+000

9.6091890e+000 3.4848812e+000 9.5173273e+000

1.0047723e+001 3.3647203e+000 9.3888941e+000

1.0620536e+001 3.2822027e+000 9.2215528e+000

9.3287258e+000 4.0657535e+000 9.9880447e+000

9.4012165e+000 3.8980458e+000 9.6415529e+000

9.6049051e+000 3.7852612e+000 9.3404589e+000

1.0173357e+001 3.5512366e+000 9.3216810e+000

1.0802080e+001 3.4880145e+000 9.1784029e+000

9.4319391e+000 4.2702355e+000 9.9086494e+000

9.5681238e+000 4.1872005e+000 9.6198101e+000

9.8755560e+000 4.0293670e+000 9.4497929e+000

1.0253578e+001 3.9018633e+000 9.3168030e+000

1.0796681e+001 3.7388895e+000 9.2690496e+000

9.6476135e+000 4.4143662e+000 9.8039761e+000

9.8956022e+000 4.4873595e+000 9.6666851e+000

1.0146400e+001 4.4549546e+000 9.5575275e+000

1.0384012e+001 4.3353014e+000 9.4488935e+000

1.0614162e+001 4.1214170e+000 9.3519011e+000

'Coimbre24

1.3301158e+000 -9.7666129e-002 1.9666524e+000

1.1460315e+000 -4.3464115e-001 2.3103206e+000

9.8936838e-001 -9.5042586e-001 2.4351068e+000

1.2022234e+000 -1.6426544e+000 2.4586992e+000

1.4575981e+000 -2.4840693e+000 2.3117213e+000

1.6918225e+000 2.1575230e-001 1.4119980e+000

1.6556873e+000 -4.1051096e-001 1.4245816e+000

1.6469520e+000 -1.2460140e+000 1.4502469e+000

1.6792519e+000 -2.2939737e+000 1.5262111e+000

1.9400191e+000 -3.4467893e+000 1.9053195e+000

2.0477757e+000 3.3143386e-001 9.3071127e-001

2.1601806e+000 -4.1203129e-001 7.6064438e-001

2.2194996e+000 -1.3808556e+000 6.4514112e-001

2.3107233e+000 -2.4854300e+000 9.1808909e-001

2.4031951e+000 -3.7603719e+000 1.4582797e+000

2.4111414e+000 2.5218838e-001 5.3762925e-001

2.6774848e+000 -4.4025108e-001 3.1370053e-001

2.7992110e+000 -1.2785567e+000 2.8745252e-001

2.8428695e+000 -2.2412441e+000 5.4086810e-001

2.7927794e+000 -3.3855152e+000 9.7941035e-001

2.7640951e+000 -2.5549078e-002 2.1111755e-001

3.1488972e+000 -4.6411061e-001 -5.1962826e-003

3.3326907e+000 -1.0233217e+000 -4.9707524e-002

3.3021793e+000 -1.6968874e+000 5.5956502e-002

3.0599437e+000 -2.4884694e+000 3.0951509e-001

'Coimbre25

-1.0073984e-001 -4.4354768e+000 8.1719017e+000

6.6127442e-002 -4.8179770e+000 7.8563967e+000

3.8943905e-001 -5.0426006e+000 7.5820799e+000

7.9049468e-001 -5.1180043e+000 7.2558031e+000

1.2471609e+000 -5.0484943e+000 6.8489504e+000

-2.2301075e-001 -3.8605201e+000 8.3633986e+000

4.3478891e-002 -4.1945267e+000 7.9451933e+000

2.9594326e-001 -4.4160614e+000 7.4072428e+000

8.9578122e-001 -4.6063895e+000 6.8851829e+000

1.8809254e+000 -4.6988392e+000 6.4137263e+000

-1.2436682e-001 -3.4085016e+000 8.4949579e+000

7.2893634e-002 -3.5873773e+000 7.9332519e+000

3.3985996e-001 -3.7710702e+000 7.3354549e+000

1.0649590e+000 -4.0056787e+000 6.7563105e+000

2.2652831e+000 -4.2389870e+000 6.3008070e+000

1.2045483e-001 -3.0251777e+000 8.5170126e+000

3.5419297e-001 -2.9689922e+000 8.0166149e+000

6.1682129e-001 -3.0558143e+000 7.4192395e+000

1.3947358e+000 -3.2885885e+000 6.9388824e+000

2.3846636e+000 -3.6834836e+000 6.4893332e+000

4.1156691e-001 -2.6178281e+000 8.4047718e+000

8.8582236e-001 -2.3241062e+000 8.1306114e+000

1.3252044e+000 -2.2778306e+000 7.7639117e+000

1.7452761e+000 -2.4757483e+000 7.3058767e+000

2.1993656e+000 -2.9688761e+000 6.8474894e+000

'Coimbre26

1.2836743e+001 6.2443066e-001 1.0261535e+001

1.2953476e+001 3.7797335e-001 1.0294763e+001

1.3195183e+001 1.6801926e-001 1.0279667e+001

1.3478346e+001 -9.0962492e-002 1.0189792e+001

1.3842828e+001 -3.5492814e-001 1.0045316e+001

1.2989991e+001 9.2935878e-001 1.0051963e+001

1.3031883e+001 7.2479892e-001 9.7225904e+000

1.3232448e+001 3.9321563e-001 9.5413637e+000

1.3725937e+001 1.7637804e-002 9.5976868e+000

1.4375042e+001 -5.3237927e-001 9.7745495e+000

1.3191021e+001 1.1250530e+000 9.8234863e+000

1.3321447e+001 9.0994793e-001 9.4208908e+000

1.3449076e+001 6.1463428e-001 9.1226578e+000

1.4034489e+001 2.4379563e-001 9.3064547e+000

1.4677505e+001 -4.8344699e-001 9.5380306e+000

1.3477873e+001 1.1397781e+000 9.6157780e+000

1.3568150e+001 1.0367749e+000 9.2091579e+000

1.3806459e+001 7.8350294e-001 9.0083828e+000

1.4270100e+001 4.2324057e-001 9.1946068e+000

1.4719313e+001 -2.9233554e-001 9.2777548e+000

1.3704517e+001 1.2044071e+000 9.3129673e+000

1.4022949e+001 1.0832860e+000 9.2229347e+000

1.4280830e+001 9.2873389e-001 9.1284428e+000

1.4472239e+001 6.7654675e-001 9.0628424e+000

1.4605205e+001 3.0923989e-001 9.1772881e+000

'Coimbre27

9.7904568e+000 -6.1406198e+000 8.1404953e+000

1.0008739e+001 -6.2132311e+000 7.7776628e+000

1.0233592e+001 -6.1050706e+000 7.4425521e+000

1.0437288e+001 -5.8186979e+000 7.1139588e+000

1.0604362e+001 -5.3618846e+000 6.7691784e+000

9.5566025e+000 -5.8675256e+000 8.4375086e+000

9.8263845e+000 -5.8382821e+000 7.9148455e+000

1.0060975e+001 -5.6305771e+000 7.4402981e+000

1.0283115e+001 -5.2668142e+000 6.9791164e+000

1.0589700e+001 -4.6907363e+000 6.5805044e+000

9.3967419e+000 -5.5342307e+000 8.6172171e+000

9.6002178e+000 -5.3925095e+000 8.0231695e+000

9.6649742e+000 -5.1966896e+000 7.4342613e+000

9.9814558e+000 -4.7814426e+000 6.9597187e+000

1.0426584e+001 -4.1987891e+000 6.5673428e+000

9.2799444e+000 -5.1480484e+000 8.6693077e+000

9.3622322e+000 -4.8855543e+000 8.1791840e+000

9.5449476e+000 -4.5807047e+000 7.7137933e+000

9.7719622e+000 -4.2558255e+000 7.2308502e+000

1.0144761e+001 -3.8739002e+000 6.7611179e+000

9.1961050e+000 -4.7113318e+000 8.5933247e+000

9.1357832e+000 -4.2558503e+000 8.3576622e+000

9.2799292e+000 -3.9371405e+000 8.0762558e+000

9.4447412e+000 -3.7562857e+000 7.6553249e+000

9.7815781e+000 -3.7093055e+000 7.1859660e+000

'Coimbre28

1.3761829e+001 3.8708889e+000 1.5722028e+001

1.3742156e+001 4.0893292e+000 1.5706267e+001

1.3649041e+001 4.2970910e+000 1.5756761e+001

1.3489574e+001 4.4945731e+000 1.5881488e+001

1.3235376e+001 4.6791625e+000 1.6046545e+001

1.3714522e+001 3.6404030e+000 1.5798652e+001

1.3518609e+001 3.8715277e+000 1.5874181e+001

1.3311232e+001 4.1320047e+000 1.5940673e+001

1.3142355e+001 4.4518538e+000 1.6096355e+001

1.2963263e+001 4.8222394e+000 1.6304571e+001

1.3648508e+001 3.4642289e+000 1.5911140e+001

1.3349316e+001 3.6586668e+000 1.6019678e+001

1.3052703e+001 3.9011431e+000 1.6055941e+001

1.2931778e+001 4.2983694e+000 1.6295042e+001

1.2802700e+001 4.7630544e+000 1.6513899e+001

1.3548856e+001 3.3335581e+000 1.6036724e+001

1.3293133e+001 3.4645488e+000 1.6225761e+001

1.3051061e+001 3.6850514e+000 1.6356256e+001

1.2813913e+001 4.0108719e+000 1.6451319e+001

1.2748117e+001 4.5000920e+000 1.6671272e+001

1.3443228e+001 3.2649972e+000 1.6211315e+001

1.3257375e+001 3.2661626e+000 1.6423580e+001

1.3100019e+001 3.3972716e+000 1.6596148e+001

1.2951414e+001 3.6541266e+000 1.6715977e+001

1.2814516e+001 4.0375819e+000 1.6785147e+001

'Coimbre29

1.1467192e+001 -4.1586709e+000 1.5187121e+001

1.1302598e+001 -4.2951617e+000 1.5444776e+001

1.1231724e+001 -4.5173950e+000 1.5682504e+001

1.1244380e+001 -4.8241873e+000 1.5892161e+001

1.1299302e+001 -5.2425280e+000 1.6035049e+001

1.1746518e+001 -4.1364775e+000 1.4914699e+001

1.1561222e+001 -4.1665592e+000 1.5060830e+001

1.1465994e+001 -4.4273386e+000 1.5310351e+001

1.1356849e+001 -4.9774261e+000 1.5584626e+001

1.1346226e+001 -5.7710786e+000 1.6008360e+001

1.1897236e+001 -4.2235174e+000 1.4659271e+001

1.1788218e+001 -4.2602072e+000 1.4768128e+001

1.1593823e+001 -4.5866814e+000 1.4914284e+001

1.1528176e+001 -5.1817932e+000 1.5294243e+001

1.1576220e+001 -6.0258660e+000 1.5878952e+001

1.2031059e+001 -4.4377918e+000 1.4502498e+001

1.1993472e+001 -4.5627241e+000 1.4573304e+001

1.1899722e+001 -4.8869615e+000 1.4738836e+001

1.1820825e+001 -5.4091229e+000 1.5058794e+001

1.1718652e+001 -6.1457410e+000 1.5535323e+001

1.2162995e+001 -4.7694759e+000 1.4455863e+001

1.2216101e+001 -5.0857215e+000 1.4500193e+001

1.2203094e+001 -5.4013028e+000 1.4611481e+001

1.2165454e+001 -5.6886311e+000 1.4848324e+001

1.2027856e+001 -5.9992461e+000 1.5096094e+001

'Coimbre30

1.5114460e+001 -5.3787165e+000 1.0568977e+001

1.5010360e+001 -5.0841894e+000 1.0625465e+001

1.4872714e+001 -4.8522224e+000 1.0729847e+001

1.4745033e+001 -4.6733832e+000 1.0914522e+001

1.4554133e+001 -4.5684962e+000 1.1128160e+001

1.5126811e+001 -5.7493792e+000 1.0640490e+001

1.5063516e+001 -5.6148114e+000 1.0720505e+001

1.4836642e+001 -5.3816290e+000 1.0824690e+001

1.4656367e+001 -5.0535779e+000 1.1117412e+001

1.4318245e+001 -4.6306424e+000 1.1452196e+001

1.5070292e+001 -6.0248218e+000 1.0775849e+001

1.4988882e+001 -5.9863353e+000 1.0876644e+001

1.4788730e+001 -5.7685857e+000 1.1041912e+001

1.4575115e+001 -5.3760376e+000 1.1342405e+001

1.4175207e+001 -4.8072319e+000 1.1684864e+001

1.4940728e+001 -6.2059207e+000 1.0971810e+001

1.4811847e+001 -6.1970210e+000 1.1116677e+001

1.4623910e+001 -6.0079374e+000 1.1299635e+001

1.4449725e+001 -5.6401739e+000 1.1564676e+001

1.4156549e+001 -5.0870667e+000 1.1836518e+001

1.4743952e+001 -6.2914405e+000 1.1232862e+001

1.4570460e+001 -6.2486420e+000 1.1463433e+001

1.4376210e+001 -6.1012650e+000 1.1624517e+001

1.4341372e+001 -5.8411875e+000 1.1821426e+001

1.4208229e+001 -5.4883409e+000 1.1885214e+001

'Coimbre31

2.1653246e+001 1.8873556e+000 1.3622914e+001

2.1605700e+001 1.7076273e+000 1.3613675e+001

2.1587002e+001 1.5236516e+000 1.3544460e+001

2.1564318e+001 1.3481847e+000 1.3404295e+001

2.1577240e+001 1.1652622e+000 1.3204943e+001

2.1664387e+001 2.0599599e+000 1.3488636e+001

2.1572533e+001 1.8988801e+000 1.3407492e+001

2.1453552e+001 1.7078471e+000 1.3256273e+001

2.1474350e+001 1.4346411e+000 1.3072753e+001

2.1676779e+001 1.1066332e+000 1.2892835e+001

2.1686310e+001 2.1906810e+000 1.3336220e+001

2.1598751e+001 2.0596554e+000 1.3198814e+001

2.1528862e+001 1.8584571e+000 1.3029167e+001

2.1572386e+001 1.5539938e+000 1.2837659e+001

2.1786098e+001 1.1706302e+000 1.2672424e+001

2.1777020e+001 2.2589333e+000 1.3169627e+001

2.1759428e+001 2.1651585e+000 1.3012733e+001

2.1768604e+001 1.9931993e+000 1.2862011e+001

2.1790207e+001 1.7212970e+000 1.2689558e+001

2.1895678e+001 1.3550736e+000 1.2539471e+001

2.1878630e+001 2.2932570e+000 1.2979845e+001

2.1966717e+001 2.2369261e+000 1.2790871e+001

2.2039379e+001 2.1070309e+000 1.2665976e+001

2.2056473e+001 1.9139692e+000 1.2572122e+001

2.2008717e+001 1.6606922e+000 1.2495419e+001

'Coimbre32

2.7614885e+001 5.0060139e+000 1.7960882e+001

2.7630062e+001 5.2295275e+000 1.7721016e+001

2.7589560e+001 5.5114608e+000 1.7581779e+001

2.7479618e+001 5.8513165e+000 1.7542561e+001

2.7363218e+001 6.2592072e+000 1.7617550e+001

2.7535971e+001 4.9180493e+000 1.8262697e+001

2.7384678e+001 5.2649126e+000 1.8250673e+001

2.7238977e+001 5.6328731e+000 1.8154560e+001

2.7210562e+001 6.0826888e+000 1.8048588e+001

2.7287039e+001 6.6096559e+000 1.7935820e+001

2.7448374e+001 4.9349079e+000 1.8512192e+001

2.7129131e+001 5.2910557e+000 1.8573563e+001

2.6916073e+001 5.6883759e+000 1.8486570e+001

2.6974457e+001 6.1751947e+000 1.8402760e+001

2.7152462e+001 6.7294383e+000 1.8193956e+001

2.7266668e+001 5.0068231e+000 1.8658205e+001

2.6934162e+001 5.3329964e+000 1.8718178e+001

2.6754845e+001 5.7018681e+000 1.8665884e+001

2.6788160e+001 6.1347380e+000 1.8578957e+001

2.6988930e+001 6.6201138e+000 1.8398626e+001

2.7029900e+001 5.1570387e+000 1.8728287e+001

2.6834158e+001 5.4008346e+000 1.8719875e+001

2.6729229e+001 5.6700664e+000 1.8688812e+001

2.6715643e+001 5.9643545e+000 1.8635340e+001

2.6786198e+001 6.2811246e+000 1.8547264e+001

'Coimbre33

1.9086344e+001 1.1027192e+000 1.2527552e+001

1.9141947e+001 1.0017442e+000 1.2369125e+001

1.9230379e+001 9.5263064e-001 1.2153040e+001

1.9377941e+001 9.5527011e-001 1.1891190e+001

1.9572479e+001 1.0097609e+000 1.1578003e+001

1.9057772e+001 1.2783856e+000 1.2661632e+001

1.9092337e+001 1.2036691e+000 1.2564584e+001

1.9167019e+001 1.1713083e+000 1.2257890e+001

1.9438564e+001 1.1563760e+000 1.1808965e+001

1.9851862e+001 1.1653557e+000 1.1193217e+001

1.9079960e+001 1.4518561e+000 1.2728079e+001

1.9099573e+001 1.4242467e+000 1.2645673e+001

1.9169891e+001 1.4182669e+000 1.2304662e+001

1.9487507e+001 1.3900857e+000 1.1791896e+001

1.9963961e+001 1.3564948e+000 1.1064691e+001

1.9145128e+001 1.6267194e+000 1.2724524e+001

1.9195210e+001 1.6664270e+000 1.2627148e+001

1.9283434e+001 1.6881680e+000 1.2315476e+001

1.9568550e+001 1.6506803e+000 1.1864757e+001

1.9939495e+001 1.5773731e+000 1.1211933e+001

1.9238604e+001 1.8070059e+000 1.2643856e+001

1.9337799e+001 1.9414014e+000 1.2487422e+001

1.9437664e+001 1.9953295e+000 1.2255119e+001

1.9585276e+001 1.9573350e+000 1.1970968e+001

1.9769728e+001 1.8297052e+000 1.1629548e+001

'Coimbre34

1.5133834e+001 -8.2830687e+000 8.8235912e+000

1.5047200e+001 -8.3186779e+000 8.8681307e+000

1.4963551e+001 -8.3653088e+000 8.8894167e+000

1.4875751e+001 -8.4058552e+000 8.8879881e+000

1.4785864e+001 -8.4454107e+000 8.8637228e+000

1.5217607e+001 -8.2774506e+000 8.7236156e+000

1.5101631e+001 -8.2949705e+000 8.7273302e+000

1.4972141e+001 -8.3402576e+000 8.7390280e+000

1.4826254e+001 -8.4084463e+000 8.7587595e+000

1.4662729e+001 -8.5009918e+000 8.7857637e+000

1.5247693e+001 -8.2954321e+000 8.6314917e+000

1.5119603e+001 -8.3123856e+000 8.6096582e+000

1.4968432e+001 -8.3426323e+000 8.6103048e+000

1.4805881e+001 -8.4271212e+000 8.6431427e+000

1.4621936e+001 -8.5401440e+000 8.7016611e+000

1.5221667e+001 -8.3249178e+000 8.5454187e+000

1.5096762e+001 -8.3494682e+000 8.5095921e+000

1.4960123e+001 -8.3918028e+000 8.5072365e+000

1.4811500e+001 -8.4556417e+000 8.5396328e+000

1.4653545e+001 -8.5471001e+000 8.6090879e+000

1.5139535e+001 -8.3658266e+000 8.4645061e+000

1.5036109e+001 -8.4194479e+000 8.4274950e+000

1.4936168e+001 -8.4595289e+000 8.4203386e+000

1.4840492e+001 -8.4890537e+000 8.4448500e+000

1.4753224e+001 -8.5154419e+000 8.5041924e+000

'Coimbre35

1.2400150e+001 -1.3224187e+000 8.2214603e+000

1.2476272e+001 -1.3200127e+000 8.1414499e+000

1.2549707e+001 -1.2877136e+000 8.0657692e+000

1.2618017e+001 -1.2268384e+000 7.9805470e+000

1.2695848e+001 -1.1294856e+000 7.9359989e+000

1.2335498e+001 -1.2699453e+000 8.2990398e+000

1.2260365e+001 -1.2173177e+000 8.1188679e+000

1.2335693e+001 -1.1396635e+000 8.0387058e+000

1.2499018e+001 -1.0623018e+000 7.9885159e+000

1.2762435e+001 -9.7099054e-001 7.9521122e+000

1.2307082e+001 -1.1983689e+000 8.3613806e+000

1.2233992e+001 -1.1068929e+000 8.2274532e+000

1.2250559e+001 -1.0172163e+000 8.0819254e+000

1.2455476e+001 -9.5871079e-001 8.0743551e+000

1.2771041e+001 -8.5940194e-001 8.0064573e+000

1.2290013e+001 -1.1100949e+000 8.4024754e+000

1.2293465e+001 -1.0336866e+000 8.3189754e+000

1.2324219e+001 -9.5494926e-001 8.2383318e+000

1.2486363e+001 -8.8805205e-001 8.1843576e+000

1.2725019e+001 -7.9624176e-001 8.0992346e+000

1.2328463e+001 -1.0076927e+000 8.4301939e+000

1.2375453e+001 -9.0773052e-001 8.3906651e+000

1.2458188e+001 -8.4998685e-001 8.3507080e+000

1.2538538e+001 -8.0572325e-001 8.2958136e+000

1.2608249e+001 -7.7098078e-001 8.2146130e+000

'Coimbre36

7.0047255e+000 5.8778834e+000 1.1474934e+001

7.0400853e+000 5.7885799e+000 1.1414866e+001

7.0969620e+000 5.7303367e+000 1.1333477e+001

7.1913567e+000 5.7098646e+000 1.1241484e+001

7.2957911e+000 5.7139277e+000 1.1122559e+001

6.9963527e+000 5.9966040e+000 1.1485021e+001

7.0090070e+000 5.9290509e+000 1.1361879e+001

7.0338979e+000 5.8702526e+000 1.1221506e+001

7.1603618e+000 5.8204088e+000 1.1105919e+001

7.3818607e+000 5.7852755e+000 1.1014570e+001

7.0169306e+000 6.0965910e+000 1.1471323e+001

7.0384374e+000 6.0477772e+000 1.1325461e+001

7.0399523e+000 6.0073223e+000 1.1163054e+001

7.1818957e+000 5.9494724e+000 1.1047688e+001

7.4342699e+000 5.8818393e+000 1.0980057e+001

7.0570426e+000 6.1801147e+000 1.1428317e+001

7.0721264e+000 6.1664047e+000 1.1289084e+001

7.1096559e+000 6.1408210e+000 1.1155049e+001

7.2327809e+000 6.0832086e+000 1.1054599e+001

7.4275260e+000 5.9975705e+000 1.0990768e+001

7.1196465e+000 6.2464609e+000 1.1357771e+001

7.1825171e+000 6.2790170e+000 1.1278010e+001

7.2461891e+000 6.2704854e+000 1.1199409e+001

7.3084569e+000 6.2209620e+000 1.1122463e+001

7.3627558e+000 6.1371188e+000 1.1037001e+001

'Coimbre37

9.6943588e+000 8.7343687e-001 4.2954922e+000

9.6031303e+000 9.3420327e-001 4.3625736e+000

9.5099735e+000 9.4872510e-001 4.4385691e+000

9.4079170e+000 9.1489553e-001 4.5138822e+000

9.3148861e+000 8.3839226e-001 4.6135631e+000

9.7746687e+000 7.6666313e-001 4.2510767e+000

9.6234503e+000 8.2072413e-001 4.3345647e+000

9.4882593e+000 8.2532352e-001 4.4321742e+000

9.3657074e+000 7.8456634e-001 4.5606217e+000

9.2640123e+000 6.9593620e-001 4.7314534e+000

9.8256435e+000 6.6188955e-001 4.2549181e+000

9.6332502e+000 6.9283062e-001 4.3101263e+000

9.4546242e+000 6.8453801e-001 4.3833132e+000

9.3344908e+000 6.4393800e-001 4.5441332e+000

9.2541971e+000 5.6835598e-001 4.7660007e+000

9.8396130e+000 5.5744195e-001 4.2915292e+000

9.6783342e+000 5.5350339e-001 4.3484478e+000

9.5170374e+000 5.2530396e-001 4.4114490e+000

9.3967361e+000 4.9508235e-001 4.5499463e+000

9.3234015e+000 4.6265867e-001 4.7543020e+000

9.8191137e+000 4.5384488e-001 4.3659863e+000

9.7551947e+000 3.9899322e-001 4.4447598e+000

9.6695824e+000 3.6043036e-001 4.5145650e+000

9.5720673e+000 3.5653493e-001 4.5979705e+000

9.4543324e+000 3.7428859e-001 4.6798410e+000

'Coimbre38

2.4412873e+000 -9.7506994e-001 1.6537172e+001

2.5118003e+000 -1.0034126e+000 1.6498650e+001

2.5982220e+000 -1.0219897e+000 1.6453728e+001

2.6993954e+000 -1.0310594e+000 1.6400774e+001

2.8100691e+000 -1.0348604e+000 1.6331350e+001

2.3718829e+000 -8.9599299e-001 1.6559860e+001

2.4494214e+000 -8.9997780e-001 1.6490938e+001

2.5665772e+000 -9.2053109e-001 1.6396524e+001

2.7414947e+000 -9.4272929e-001 1.6316355e+001

2.9695508e+000 -9.7284120e-001 1.6231112e+001

2.3463697e+000 -8.1340420e-001 1.6556282e+001

2.4253576e+000 -8.0221623e-001 1.6462946e+001

2.5514224e+000 -8.1908262e-001 1.6332838e+001

2.7595994e+000 -8.4273916e-001 1.6257833e+001

3.0361009e+000 -8.8132423e-001 1.6199488e+001

2.3736727e+000 -7.2295010e-001 1.6544849e+001

2.4627321e+000 -6.9647676e-001 1.6453535e+001

2.5819123e+000 -6.9745654e-001 1.6333662e+001

2.7685878e+000 -7.2213340e-001 1.6268845e+001

2.9996166e+000 -7.7163619e-001 1.6199089e+001

2.4361310e+000 -6.2946749e-001 1.6491215e+001

2.5419896e+000 -5.7902700e-001 1.6432323e+001

2.6440337e+000 -5.6254053e-001 1.6364674e+001

2.7472579e+000 -5.8157051e-001 1.6293066e+001

2.8627400e+000 -6.3992935e-001 1.6239378e+001

'Coimbre39

1.0276912e+001 -7.1372575e-001 1.1707555e+001

1.0330549e+001 -7.0023161e-001 1.1666494e+001

1.0378222e+001 -6.7546684e-001 1.1628803e+001

1.0418725e+001 -6.4105660e-001 1.1593166e+001

1.0450980e+001 -5.9939432e-001 1.1555436e+001

1.0249105e+001 -6.6561216e-001 1.1769107e+001

1.0280908e+001 -6.6654563e-001 1.1653621e+001

1.0341431e+001 -6.2291878e-001 1.1595879e+001

1.0403029e+001 -5.7075304e-001 1.1553611e+001

1.0460279e+001 -5.1920754e-001 1.1493732e+001

1.0230119e+001 -6.0522193e-001 1.1794488e+001

1.0244825e+001 -6.0720241e-001 1.1660346e+001

1.0294557e+001 -5.7311869e-001 1.1572990e+001

1.0365105e+001 -5.1820242e-001 1.1524505e+001

1.0436293e+001 -4.6431175e-001 1.1464218e+001

1.0186104e+001 -5.6975824e-001 1.1766022e+001

1.0213334e+001 -5.3752887e-001 1.1671580e+001

1.0261780e+001 -4.9760020e-001 1.1608184e+001

1.0319250e+001 -4.5981312e-001 1.1546247e+001

1.0383411e+001 -4.2802843e-001 1.1478028e+001

1.0163414e+001 -5.1422495e-001 1.1709863e+001

1.0178922e+001 -4.6428007e-001 1.1679824e+001

1.0209910e+001 -4.2819947e-001 1.1644167e+001

1.0253385e+001 -4.0786228e-001 1.1599103e+001

1.0306526e+001 -4.0606689e-001 1.1540603e+001

'Coimbre40

9.1818066e+000 -5.1042924e+000 8.1953497e+000

9.0892925e+000 -4.9486532e+000 8.2185774e+000

8.9978189e+000 -4.8332586e+000 8.2415848e+000

8.9083529e+000 -4.7657599e+000 8.3403320e+000

8.8240299e+000 -4.7498412e+000 8.4979439e+000

9.2102385e+000 -5.3080306e+000 8.2244520e+000

8.9825640e+000 -5.2038527e+000 8.1929998e+000

8.8247938e+000 -5.1068039e+000 8.2402096e+000

8.6943312e+000 -4.9955854e+000 8.3472586e+000

8.6426191e+000 -4.8380113e+000 8.6386976e+000

9.2117939e+000 -5.4095201e+000 8.3367090e+000

8.9501448e+000 -5.3475399e+000 8.2842093e+000

8.7245560e+000 -5.3090868e+000 8.2869577e+000

8.6261358e+000 -5.1574793e+000 8.4662771e+000

8.6393747e+000 -4.9255366e+000 8.7949686e+000

9.1790981e+000 -5.4625525e+000 8.4622307e+000

8.9969292e+000 -5.4028454e+000 8.4806919e+000

8.7949123e+000 -5.3737864e+000 8.5160389e+000

8.7551613e+000 -5.1924233e+000 8.7051306e+000

8.6404896e+000 -5.0671687e+000 8.8483257e+000

9.1068192e+000 -5.4682746e+000 8.6131830e+000

8.9597979e+000 -5.4552212e+000 8.7226868e+000

8.8800621e+000 -5.3987241e+000 8.8145885e+000

8.7953548e+000 -5.3200264e+000 8.8619080e+000

8.7238207e+000 -5.2130027e+000 8.8694410e+000

'Coimbre41

1.6238514e+001 1.2452167e+001 1.3227162e+001

1.6361956e+001 1.1854152e+001 1.3300018e+001

1.6704704e+001 1.1203289e+001 1.3247628e+001

1.7255878e+001 1.0497293e+001 1.3051834e+001

1.8011429e+001 9.7364168e+000 1.2707993e+001

1.6502533e+001 1.3275234e+001 1.2884716e+001

1.6322517e+001 1.2895417e+001 1.3068567e+001

1.6512001e+001 1.1943589e+001 1.2734924e+001

1.7647533e+001 1.0685247e+001 1.2385160e+001

1.9530867e+001 9.0858507e+000 1.1763161e+001

1.6822651e+001 1.3780720e+001 1.2550335e+001

1.6622934e+001 1.3596327e+001 1.2710880e+001

1.6438000e+001 1.2419345e+001 1.2125526e+001

1.7893459e+001 1.1049638e+001 1.1782012e+001

2.0290388e+001 9.1785460e+000 1.1233989e+001

1.7184263e+001 1.3964384e+001 1.2179760e+001

1.6919773e+001 1.3691157e+001 1.2215833e+001

1.7018595e+001 1.2763786e+001 1.1724580e+001

1.8450914e+001 1.1679568e+001 1.1660846e+001

2.0347696e+001 1.0011906e+001 1.1224947e+001

1.7697266e+001 1.3873883e+001 1.1985144e+001

1.8051033e+001 1.3612984e+001 1.1853754e+001

1.8498283e+001 1.3152875e+001 1.1790866e+001

1.9009411e+001 1.2475386e+001 1.1661598e+001

1.9601398e+001 1.1590954e+001 1.1530909e+001

'Coimbre42

2.2447817e+001 1.9339617e+000 2.2597607e+001

2.2266680e+001 1.7369488e+000 2.2749537e+001

2.2201181e+001 1.5485746e+000 2.2895296e+001

2.2174921e+001 1.3510276e+000 2.2946947e+001

2.2229969e+001 1.1504173e+000 2.2946770e+001

2.2648300e+001 1.9660785e+000 2.2417124e+001

2.2492434e+001 1.8512290e+000 2.2551058e+001

2.2367558e+001 1.6203251e+000 2.2664341e+001

2.2290445e+001 1.2832544e+000 2.2790726e+001

2.2304335e+001 8.5174870e-001 2.2964882e+001

2.2812683e+001 1.9150264e+000 2.2295649e+001

2.2686668e+001 1.8234419e+000 2.2397715e+001

2.2548954e+001 1.5727638e+000 2.2487022e+001

2.2482969e+001 1.2152238e+000 2.2688856e+001

2.2400263e+001 7.0308840e-001 2.2891186e+001

2.2961555e+001 1.7868993e+000 2.2264074e+001

2.2895100e+001 1.6647924e+000 2.2343439e+001

2.2803263e+001 1.4406781e+000 2.2446468e+001

2.2685104e+001 1.1112688e+000 2.2562576e+001

2.2540972e+001 7.0662618e-001 2.2734165e+001

2.3070915e+001 1.5735390e+000 2.2292425e+001

2.3101524e+001 1.3635743e+000 2.2358440e+001

2.3060278e+001 1.1700244e+000 2.2413361e+001

2.2955166e+001 1.0039515e+000 2.2467016e+001

2.2810484e+001 8.6517704e-001 2.2577330e+001

'Coimbre43

1.4460883e+001 7.3444633e+000 9.0435343e+000

1.4582106e+001 7.3759294e+000 8.8232994e+000

1.4704493e+001 7.4897499e+000 8.6597900e+000

1.4799582e+001 7.6727839e+000 8.5249271e+000

1.4877528e+001 7.9296346e+000 8.4288750e+000

1.4336662e+001 7.4335413e+000 9.2339611e+000

1.4389446e+001 7.4012218e+000 9.1257811e+000

1.4446376e+001 7.5070691e+000 8.9053335e+000

1.4627167e+001 7.8140459e+000 8.6503515e+000

1.4883054e+001 8.3060398e+000 8.3178005e+000

1.4251040e+001 7.5668263e+000 9.3710632e+000

1.4268751e+001 7.5483160e+000 9.3271561e+000

1.4242999e+001 7.6309576e+000 9.0581322e+000

1.4455145e+001 7.9758644e+000 8.7572470e+000

1.4812628e+001 8.5299654e+000 8.3614874e+000

1.4170502e+001 7.7314587e+000 9.4425497e+000

1.4168463e+001 7.7781405e+000 9.4065933e+000

1.4163879e+001 7.9095802e+000 9.2006807e+000

1.4283646e+001 8.1737366e+000 8.8746910e+000

1.4650753e+001 8.5987377e+000 8.5423040e+000

1.4067138e+001 7.9195142e+000 9.4380245e+000

1.4107485e+001 8.1462202e+000 9.4047394e+000

1.4164803e+001 8.3199482e+000 9.2916384e+000

1.4204911e+001 8.4433508e+000 9.0850801e+000

1.4375000e+001 8.5088024e+000 8.8419552e+000

'Coimbre44

1.6120850e+001 8.1601763e+000 6.5769696e+000

1.6064611e+001 8.2155809e+000 6.6521711e+000

1.6019556e+001 8.2427120e+000 6.7359638e+000

1.5966707e+001 8.2454367e+000 6.8142610e+000

1.5913600e+001 8.2223206e+000 6.8925600e+000

1.6162685e+001 8.0716543e+000 6.5422106e+000

1.6121418e+001 8.0973005e+000 6.5708351e+000

1.6052486e+001 8.1207933e+000 6.6498342e+000

1.5985792e+001 8.1417379e+000 6.7960014e+000

1.5858139e+001 8.1591187e+000 6.9764733e+000

1.6175789e+001 7.9895134e+000 6.5375199e+000

1.6147285e+001 7.9916234e+000 6.5554113e+000

1.6063444e+001 8.0064716e+000 6.6289682e+000

1.5982450e+001 8.0383177e+000 6.7912560e+000

1.5838497e+001 8.0801029e+000 7.0099807e+000

1.6154676e+001 7.9123468e+000 6.5577579e+000

1.6124092e+001 7.8947940e+000 6.5961084e+000

1.6061230e+001 7.9008718e+000 6.6774402e+000

1.5975675e+001 7.9315844e+000 6.8125052e+000

1.5860532e+001 7.9859076e+000 6.9974761e+000

1.6122627e+001 7.8434334e+000 6.6266236e+000

1.6077257e+001 7.8069205e+000 6.7085476e+000

1.6028244e+001 7.8003001e+000 6.7872753e+000

1.5973731e+001 7.8229804e+000 6.8616910e+000

1.5916867e+001 7.8757734e+000 6.9336758e+000

'Coimbre45

1.7091194e+001 8.5779983e-001 9.5119295e+000

1.7051641e+001 5.8334631e-001 9.5608377e+000

1.7042536e+001 2.9035589e-001 9.5460358e+000

1.7087292e+001 -2.2298729e-002 9.4826241e+000

1.7141630e+001 -3.5302362e-001 9.3406200e+000

1.7188408e+001 1.0901773e+000 9.2882891e+000

1.7136965e+001 7.2223556e-001 9.3278913e+000

1.7094959e+001 3.0459124e-001 9.2837925e+000

1.7164055e+001 -1.7407608e-001 9.2235870e+000

1.7299435e+001 -7.1114862e-001 9.1157713e+000

1.7325043e+001 1.1715484e+000 9.0775385e+000

1.7307674e+001 7.7233082e-001 9.1071329e+000

1.7220282e+001 3.1636372e-001 9.0055761e+000

1.7311834e+001 -2.1966662e-001 8.9800014e+000

1.7418392e+001 -8.1666440e-001 8.9003000e+000

1.7494907e+001 1.1082807e+000 8.8815985e+000

1.7499357e+001 7.4036032e-001 8.8422756e+000

1.7462383e+001 3.2248551e-001 8.7479820e+000

1.7513035e+001 -1.5638624e-001 8.7399015e+000

1.7564896e+001 -6.8325728e-001 8.7294350e+000

1.7647324e+001 8.9620751e-001 8.6544952e+000

1.7743582e+001 6.2090224e-001 8.5313101e+000

1.7775499e+001 3.2909939e-001 8.4627314e+000

1.7773539e+001 1.7985202e-002 8.4965639e+000

1.7709545e+001 -3.0463856e-001 8.5858421e+000

'Coimbre46

1.6531891e+001 2.0937626e+000 9.9300919e+000

1.6529535e+001 1.9021066e+000 1.0001728e+001

1.6541746e+001 1.7019986e+000 1.0020547e+001

1.6579466e+001 1.4935478e+000 9.9911633e+000

1.6653898e+001 1.2763838e+000 9.9198742e+000

1.6628347e+001 2.2528977e+000 9.7870512e+000

1.6619162e+001 1.9755906e+000 9.8170815e+000

1.6622873e+001 1.6868739e+000 9.8128443e+000

1.6685177e+001 1.3887765e+000 9.8006630e+000

1.6815718e+001 1.0892991e+000 9.7892485e+000

1.6749289e+001 2.3160620e+000 9.6594267e+000

1.6738077e+001 2.0177932e+000 9.6451025e+000

1.6753351e+001 1.7041402e+000 9.6279469e+000

1.6819101e+001 1.3749293e+000 9.6262798e+000

1.6953438e+001 1.0476513e+000 9.6598244e+000

1.6862833e+001 2.2843518e+000 9.5282497e+000

1.6886423e+001 2.0277426e+000 9.4845333e+000

1.6928337e+001 1.7548554e+000 9.4719706e+000

1.6950218e+001 1.4409875e+000 9.4427586e+000

1.7025932e+001 1.1234710e+000 9.4888487e+000

1.6973526e+001 2.1575062e+000 9.3929052e+000

1.7060390e+001 2.0054407e+000 9.3238468e+000

1.7108482e+001 1.8218921e+000 9.2922506e+000

1.7113539e+001 1.6060166e+000 9.2915297e+000

1.7085348e+001 1.3601519e+000 9.3355322e+000

'Coimbre47

1.7143579e+001 9.6970993e-001 9.4172630e+000

1.7065834e+001 7.7127057e-001 9.5301561e+000

1.7037760e+001 5.0286627e-001 9.5600719e+000

1.7063810e+001 1.6401808e-001 9.5097857e+000

1.7139095e+001 -2.4474005e-001 9.3758097e+000

1.7231470e+001 1.1090366e+000 9.1781273e+000

1.7165596e+001 7.7283669e-001 9.3070421e+000

1.7085188e+001 3.6562756e-001 9.3009729e+000

1.7159187e+001 -1.1992907e-001 9.2858801e+000

1.7251265e+001 -6.7315674e-001 9.1707697e+000

1.7393137e+001 1.1417477e+000 8.9875221e+000

1.7329542e+001 7.5692213e-001 9.0746946e+000

1.7216515e+001 2.9598716e-001 9.0112581e+000

1.7282089e+001 -2.3252723e-001 9.0172205e+000

1.7377514e+001 -8.2516283e-001 8.9684086e+000

1.7567699e+001 1.0766295e+000 8.8114767e+000

1.7531511e+001 7.1421051e-001 8.7852316e+000

1.7480326e+001 2.9518560e-001 8.7293930e+000

1.7505316e+001 -1.8007669e-001 8.7521095e+000

1.7520657e+001 -7.0112008e-001 8.7682180e+000

1.7717237e+001 9.1808861e-001 8.5917006e+000

1.7822977e+001 6.5404946e-001 8.4787941e+000

1.7848419e+001 3.6289284e-001 8.4290218e+000

1.7808538e+001 4.1918073e-002 8.4639864e+000

1.7705929e+001 -3.0993733e-001 8.5879269e+000

'Coimbre48

1.7209772e+001 1.3093897e+000 9.2301092e+000

1.7238623e+001 1.5574975e+000 9.1855106e+000

1.7245686e+001 1.7977661e+000 9.1728020e+000

1.7216976e+001 2.0296898e+000 9.1762648e+000

1.7163578e+001 2.2543471e+000 9.2092972e+000

1.7109793e+001 1.1186826e+000 9.4099522e+000

1.7131868e+001 1.4287419e+000 9.2763033e+000

1.7164541e+001 1.7683758e+000 9.2544708e+000

1.7111427e+001 2.1267142e+000 9.2851057e+000

1.7011202e+001 2.5131674e+000 9.3813686e+000

1.6984756e+001 1.0424881e+000 9.5900784e+000

1.6967270e+001 1.3685281e+000 9.4403067e+000

1.6966089e+001 1.7423694e+000 9.3942146e+000

1.6930410e+001 2.1517906e+000 9.4350471e+000

1.6850397e+001 2.6017110e+000 9.5493879e+000

1.6834108e+001 1.0805016e+000 9.7700882e+000

1.6770039e+001 1.3823861e+000 9.6843567e+000

1.6727139e+001 1.7249433e+000 9.6454172e+000

1.6729597e+001 2.1055832e+000 9.6805477e+000

1.6680059e+001 2.5186913e+000 9.7232227e+000

1.6630941e+001 1.2269499e+000 9.9406319e+000

1.6554777e+001 1.4703846e+000 1.0028674e+001

1.6512417e+001 1.7237566e+000 1.0050846e+001

1.6507999e+001 1.9856918e+000 1.0015245e+001

1.6564449e+001 2.2615469e+000 9.9392033e+000

'Coimbre49

1.6316360e+001 2.7930112e+000 1.0100011e+001

1.6439259e+001 2.8652635e+000 9.9266987e+000

1.6523975e+001 3.0011811e+000 9.7877483e+000

1.6584421e+001 3.2042506e+000 9.6987886e+000

1.6589262e+001 3.4670129e+000 9.6231012e+000

1.6173664e+001 2.8212178e+000 1.0312386e+001

1.6147335e+001 2.9630678e+000 1.0293396e+001

1.6191450e+001 3.1694014e+000 1.0162482e+001

1.6299191e+001 3.4389307e+000 9.9101028e+000

1.6533607e+001 3.7914138e+000 9.5933571e+000

1.6066454e+001 2.9164987e+000 1.0481333e+001

1.5966433e+001 3.1151295e+000 1.0541212e+001

1.5964855e+001 3.3520176e+000 1.0407044e+001

1.6119635e+001 3.6455419e+000 1.0118203e+001

1.6406998e+001 3.9883275e+000 9.6581612e+000

1.5957776e+001 3.0647299e+000 1.0584645e+001

1.5862025e+001 3.3078940e+000 1.0646317e+001

1.5853667e+001 3.5501680e+000 1.0526916e+001

1.5972440e+001 3.8025026e+000 1.0254507e+001

1.6240484e+001 4.0676703e+000 9.8437338e+000

1.5870788e+001 3.2738531e+000 1.0637661e+001

1.5825761e+001 3.5428803e+000 1.0603882e+001

1.5818514e+001 3.7570052e+000 1.0495998e+001

1.5895719e+001 3.9175556e+000 1.0349648e+001

1.6016689e+001 4.0248799e+000 1.0136236e+001

'Coimbre50

1.4202862e+001 -2.2744269e+000 1.4520884e+001

1.4270710e+001 -1.9668736e+000 1.4535188e+001

1.4244570e+001 -1.6430416e+000 1.4690500e+001

1.4132724e+001 -1.3126652e+000 1.5008550e+001

1.3931005e+001 -9.7073472e-001 1.5479373e+001

1.4018916e+001 -2.5287337e+000 1.4574179e+001

1.4159968e+001 -2.2438898e+000 1.4537293e+001

1.4108740e+001 -1.8209554e+000 1.4714093e+001

1.3920220e+001 -1.2624379e+000 1.5190122e+001

1.3577275e+001 -5.6026828e-001 1.5953035e+001

1.3865822e+001 -2.7152629e+000 1.4802452e+001

1.3887945e+001 -2.4407167e+000 1.4688928e+001

1.3833222e+001 -1.9924484e+000 1.4912775e+001

1.3669498e+001 -1.3671110e+000 1.5443875e+001

1.3316303e+001 -5.2391773e-001 1.6252306e+001

1.3663200e+001 -2.8262053e+000 1.5079304e+001

1.3592001e+001 -2.6117055e+000 1.5123704e+001

1.3422860e+001 -2.1876638e+000 1.5336713e+001

1.3293096e+001 -1.6124742e+000 1.5762125e+001

1.3175711e+001 -8.6606067e-001 1.6388367e+001

1.3413961e+001 -2.8634751e+000 1.5428430e+001

1.3226474e+001 -2.7357614e+000 1.5788568e+001

1.3126545e+001 -2.4840374e+000 1.6067738e+001

1.3077453e+001 -2.0955856e+000 1.6247662e+001

1.3064737e+001 -1.5689083e+000 1.6321918e+001
